# Optimization of Combined Ultrasound and Microwave-Assisted Extraction for Enhanced Bioactive Compounds Recovery from Four Medicinal Plants: Oregano, Rosemary, Hypericum, and Chamomile

**DOI:** 10.3390/molecules29235773

**Published:** 2024-12-06

**Authors:** Konstantina Theodora Laina, Christina Drosou, Chrysanthos Stergiopoulos, Panagiota Maria Eleni, Magdalini Krokida

**Affiliations:** Laboratory of Process Analysis and Design, School of Chemical Engineering, National Technical University of Athens, 9 Iroon Polytechneiou St. Zografou Campus, 15780 Athens, Greece; konstantinalaina@mail.ntua.gr (K.T.L.); chrisxp3@hotmail.com (C.S.); pneleni@gmail.com (P.M.E.); mkrok@chemeng.ntua.gr (M.K.)

**Keywords:** antioxidant activity, bioactive compounds, extraction optimization, medicinal plants, microwave-assisted extraction (MAE), oregano, phenolic compounds, rosemary, hypericum, ultrasound-assisted extraction (UAE)

## Abstract

This study presents the synergistic application of ultrasound- and microwave-assisted extraction (UAE–MAE) as a novel and efficient method for recovering bioactive compounds from the medicinal plants oregano, rosemary, *Hypericum perforatum*, and chamomile. Extraction parameters, including microwave (MW) power, ultrasound (US) power, and extraction time, were optimized using the response surface methodology (RSM), with ethanol as the solvent. Extracts were evaluated for total phenolic content (TPC) via the Folin–Ciocalteu method and antioxidant activity (IC50) using the DPPH assay. High-performance liquid chromatography with diode array detection (HPLC–DAD) identified the main bioactive compounds contributing to their antioxidant and therapeutic potential. The optimized UAE–MAE conditions enhanced phenolic recovery and antioxidant potential across all plants. Notably, *Hypericum perforatum* exhibited the highest TPC (53.7 mg GAE/g) and strongest antioxidant activity (IC50 29.8 mg extract/g) under 200 W MW, 450 W US, and 12 min, yielding 14.5%. Rosemary achieved the highest yield (23.36%) with a TPC of 26.35 mg GAE/g and an IC50 of 40.75 mg extract/g at 200 W MW, 700 W US, and 8 min. Oregano’s optimal conditions (500 W MW, 700 W US, 12 min) produced a TPC of 34.99 mg GAE/g and an IC50 of 50.31 mg extract/g. Chamomile extracts demonstrated lower phenolic content and antioxidant activity but achieved significant yields under 500 W MW, 700 W US, and 5 min. This study highlights UAE–MAE’s superior efficiency, showcasing its potential to maximize phenolic recovery sustainably, making it a promising technique for industrial and therapeutic applications.

## 1. Introduction

The growing awareness of the health benefits associated with plant-derived bioactive compounds has driven significant interest in optimizing extraction processes to make these compounds more accessible for applications in pharmaceuticals, nutraceuticals, cosmetics, and functional foods [1,2,3]. Among these bioactive compounds, phenolic compounds are particularly valued for their potent antioxidant, anti-inflammatory, and antimicrobial properties, which play a critical role in reducing oxidative stress—a major contributor to chronic diseases such as cancer, cardiovascular disorders, and neurodegenerative conditions [4,5,6,7,8]. Given the increasing prevalence of these conditions globally, there is a substantial and growing demand for bioactive compounds that offer preventive health benefits and therapeutic support [9,10]. However, optimizing extraction techniques to maximize yields of these valuable bioactives while preserving compound integrity and minimizing environmental impact remains a core challenge in the field [7]. Addressing this challenge is essential to meet the demands of various health-focused industries seeking high-quality bioactive extracts [11,12].

The selection of Mediterranean medicinal plants such as oregano (*Origanum vulgare*), rosemary (*Rosmarinus officinalis*), hypericum (*Hypericum perforatum*), and chamomile (*Matricaria recutita*) for this study was based on their well-documented bioactive properties, rich phenolic profiles, extensive traditional use, and abundance in the Mediterranean region [13,14,15,16]. These plants are not only rich in valuable compounds like polyphenols and flavonoids but also represent a largely unvalorized natural resource within the region, making their utilization both economically and environmentally sustainable [17,18,19]. As these species are readily available in Mediterranean ecosystems, their effective use supports local biodiversity while offering a low-cost, sustainable source of bioactive compounds, enhancing the value of local flora and contributing to regional bioeconomies [18,20,21]. Furthermore, these bioactive compounds are increasingly utilized as natural preservatives and functional ingredients in the food industry, where they aid in extending the shelf life and enhancing the nutritional value of products [22]. By selecting these plants, we tap into a readily available, sustainable resource that aligns with the growing demand for bioactive-rich products across various sectors.

The extraction of phenolic compounds from these plants is essential for accessing their bioactive properties [18]. Conventional extraction methods, such as Soxhlet extraction, have been widely employed for this purpose. Soxhlet extraction, while effective, is associated with significant drawbacks, including lengthy processing times, high solvent requirements, and the risk of thermal degradation of bioactive compounds [23,24,25]. These limitations not only increase production costs but also raise environmental concerns due to high solvent consumption [23,26]. Consequently, alternative “green” extraction technologies are becoming more attractive, as they promise increased efficiency, reduced environmental impact, and lower energy consumption [20,27,28,29].

Among green extraction technologies, ultrasound-assisted extraction (UAE) and microwave-assisted extraction (MAE) have garnered particular attention. UAE enhances the extraction process by generating cavitation bubbles through ultrasonic waves, which disrupt plant cell walls, facilitating the release of intracellular compounds [30,31,32]. This method offers several advantages, including shorter extraction times, reduced solvent consumption, and the preservation of heat-sensitive compounds, making it an ideal alternative to traditional methods [33,34,35,36,37,38,39]. Similarly, MAE utilizes microwave energy to achieve rapid solvent penetration and heating, accelerating extraction kinetics [39,40,41]. MAE is particularly effective in reducing extraction times and enhancing yields while maintaining energy efficiency [41,42,43,44]. Despite the promising results of UAE and MAE individually, the combination of these techniques has not been explored, especially for Mediterranean medicinal plants rich in phenolic compounds.

Recent studies have optimized UAE and MAE individually for various plant matrices. However, their synergistic application remains largely unexplored, especially for Mediterranean medicinal plants rich in bioactive compounds. While studies have demonstrated the environmental and efficiency benefits of UAE and MAE independently, combining these methods for enhanced extraction efficiency and bioactive integrity is under-researched [28,45,46,47,48,49,50]. Existing research on medicinal plants such as oregano, rosemary, hypericum, and chamomile has focused on traditional methods like Soxhlet extraction or single green techniques, neglecting dual optimization of UAE and MAE using advanced tools like the response surface methodology (RSM) [20,29,51,52,53,54,55,56].

This study utilized simultaneous ultrasound- and microwave-assisted extraction (UAE–MAE) to recover bioactive compounds from four Mediterranean medicinal plants: oregano, rosemary, *Hypericum perforatum*, and chamomile. Ethanol, an environmentally friendly solvent, was employed to enhance the solubilization of phenolic compounds. The synergistic effects of UAE and MAE were investigated, surpassing the limitations of conventional and single-mode extraction techniques. Extraction parameters, including ultrasound and microwave intensity, extraction time, and solid-to-solvent ratio, were systematically optimized using the response surface methodology (RSM) to achieve efficient extraction with reduced energy consumption and solvent usage. The optimization of the extraction process was based on the responses of the extraction yield (Y, %), the total phenolic content (TPC) and antioxidant activity of the extracts. High-performance liquid chromatography with diode array detection (HPLC–DAD) was used to identify and quantify key phenolic compounds, providing a primary compositional profile of the extracts. These compounds, known for their antioxidant and therapeutic properties, contributed to the bioactive quality of the optimized extracts. The UAE–MAE methodology aims to enhance extraction efficiency and preserved bioactive compounds while utilizing locally abundant plant materials. The optimized process will provide insights into the recovery of phenolic compounds from medicinal plants under sustainable extraction conditions.

## 2. Results and Discussion

### 2.1. Recovery of Oregano Extracts

The recovery of ethanolic extracts from oregano (*Origanum vulgare*) was examined under various conditions of ultrasound and microwave power, combined with different extraction times. Ultrasound power ranged from 450 W to 700 W, and microwave power from 200 W to 500 W, with extraction times between 5 and 12 min. The best extraction conditions for oregano ethanolic extract were obtained with 500 W microwave power and 700 W ultrasound power for 12 min, resulting in the highest yield (16.57%). This condition also displayed a strong DPPH scavenging activity (50.31 mg extract/g) and a total phenolic content of 34.99 mg GAE/g.

#### 2.1.1. Model Fitting

The table below presents the analysis of variance (ANOVA) results for the response surface models, evaluating the effects of various factors on the extraction yield (Y%), IC_50_ (mg extract/g raw material), and total phenolic content (TPC) (mg GAE/g raw material) in oregano extracts.

##### Extraction Yield

The response surface model for the extraction yield (*Y*, %) of oregano extracts is summarized in Table 1. The model demonstrated a good fit with an *R*^2^ value of 0.93, indicating that 93% of the variation in the extraction yield could be explained by the model. The statistical significance of the terms was assessed using F-values and *p*-values, with higher F-values and smaller *p*-values signifying more impactful variables.

The fitted surface model for the extraction yield (*Y*%) is given by the following equation:

(1) $$\Upsilon \left(\%\right)=7.85+1.51\times 10^{-2}X_{1}-2.54\times 10^{-5}X_{1}^{2}+1.37\times 10^{-3}\;X_{2}-1.79\times 10^{-6}X_{2}^{2}-1.28\times X_{3}\;+8.76\times 10^{-2}X_{3}^{2}-1.75\times 10^{-5}X_{1}X_{2}+1.51\times 10^{-3}X_{1}X_{3}+7.94\times 10^{-4}X_{2}X_{3}$$

Among the model terms, the linear effect of extraction time (*X*_3_) was the most significant factor influencing the extraction yield, as indicated by its F-value of 228.07 (*p* < 0.001). This demonstrates that extraction time had the strongest impact on the extraction yield of oregano. The linear term for microwave power (*X*_1_) also showed a substantial influence (F = 128.36, *p* < 0.001), suggesting that increasing microwave power enhances the extraction yield. The quadratic term for microwave power (*X*_1_^2^) was also significant (F = 14.77, *p* < 0.001), indicating a non-linear effect of microwave power on the extraction process.

In addition, the linear term for ultrasound power (*X*_2_) showed significant importance (F = 18.90, *p* < 0.001), highlighting that ultrasound power plays a role in maximizing the extraction yield. The interaction terms (*X*_1_*X*_3_ and *X*_1_*X*_2_) also had notable significance, with F-values of 35.25 (*p* < 0.001) and 47.81 (*p* < 0.001), respectively, indicating that combined effects of microwave power, ultrasound power, and extraction time further influenced the yield. Non-significant terms included the quadratic term for ultrasound power (*X*_2_^2^) (F = 0.25, *p* > 0.05) and the interaction between ultrasound power and extraction time (*X*_2_*X*_3_) (F = 19.24, *p* < 0.001).

These results suggest that extraction time, microwave power, and ultrasound power are the most critical factors influencing the extraction yield. A new regression analysis was performed, excluding the non-significant variables. The updated fitted surface model is expressed as:

(2) $$\Upsilon \left(\%\right)=7.57+1.56\times 10^{-2}X_{1}+1.65\times 10^{-4}X_{2}-1.20\times 10^{0}X_{3}-2.64\times 10^{-5}X_{1}^{2}\;+8.29\times 10^{-2}X_{3}^{2}-1.75\times 10^{-5}X_{1}X_{2}+1.51\times 10^{-3}X_{1}X_{3}+7.94\times 10^{-4}X_{2}X_{3}$$

**Figure 1:**
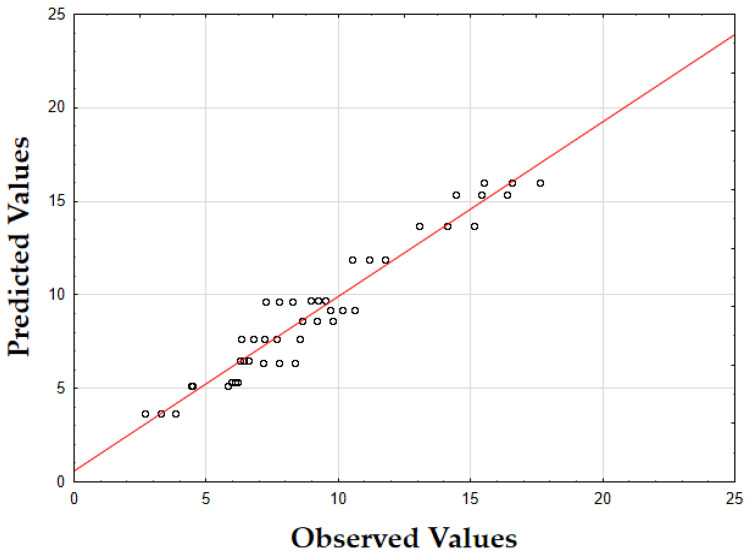
Plots of predicted versus actual values for the *Y* (%) of oregano extracts.

The F-value of 405.74 and the *p*-value of 0.00 indicate that the updated model is highly significant in predicting the extraction yield. The *R*^2^ value of 0.93 also demonstrates that the model fits the data well, accounting for most of the variability. Finally, as shown in Figure 1, the data points are tightly grouped along the diagonal, indicating very little difference between the predicted and the observed extraction yield (*Y*) values. This suggests that the errors were minimal across the full range of values, demonstrating that the model reliably and accurately predicted the extraction yield of oregano extracts.

In conclusion, extraction time (*X*_3_) was the most critical variable affecting the extraction yield (*Y*%) of oregano, as demonstrated by its high F-value and low *p*-value in the linear term. The impact of microwave power (*X*_1_) was also significant, especially in both its linear and quadratic forms, indicating direct and non-linear effects on the extraction yield. Additionally, the interaction between microwave power and ultrasound power (*X*_1_*X*_2_) had a notable influence, further emphasizing the synergistic role of these variables. These findings are supported by recent studies on *Origanum vulgare*, where multivariate optimization of ultrasound-assisted extraction demonstrated the importance of controlling sonication parameters to maximize bioactive compound recovery [57]. Moreover, research on *Opuntia fruit* peels also demonstrated the critical role of extraction time in maximizing the yield of bioactive compounds in both ultrasound- and microwave-assisted extractions, showing optimal results at shorter times to enhance phenolic and flavonoid recovery [42]. This study underscores the importance of optimizing extraction parameters, including time, microwave, and ultrasound power, to achieve high extraction yields in oregano extracts.

##### IC_50_

For IC_50_, representing antioxidant activity, the model provided a strong fit, with an *R*^2^ value of 0.97 (Table 1). The mathematical model for IC_50_ is given by the following equation:

(3) $${\mathrm{IC}}_{50}=108.41+1.34\times 10^{-1}X_{1}-3.00\times 10^{-4}X_{1}^{2}+1.04\times 10^{-1}X_{2}-1.00\times 10^{-4}X_{2}^{2}-15.44X_{3}+\;8.51\times 10^{-1}X_{3}^{2}+2.90\times 10^{-3}X_{1}X_{3}+1.00\times 10^{-4}X_{2}X_{3}$$

The quadratic term for microwave power (*X*_1_^2^) was the most significant factor influencing IC_50_**,** with an F-value of 412.74 (*p* < 0.001). This suggests that microwave power had a substantial non-linear effect on reducing IC_50_, thereby improving antioxidant activity. Additionally, the quadratic term for ultrasound power (*X*_2_^2^) was significant (F = 239.06, *p* < 0.001), indicating that ultrasound power also had a strong non-linear impact on antioxidant capacity. The linear terms for microwave power (*X*_1_) and ultrasound power (*X*_2_) had smaller but still significant effects (F = 34.37 and F = 34.12, respectively, *p* < 0.001), highlighting the importance of these parameters in optimizing antioxidant activity. The quadratic term for extraction time (*X*_3_^2^) also showed significance (F = 98.09, *p* < 0.001), demonstrating that longer extraction times enhance antioxidant capacity. In contrast, the linear term for extraction time (*X*_3_) was non-significant (F = 2.34, *p* > 0.05), indicating that changes in extraction time did not have a linear impact on antioxidant activity.

A new regression analysis, excluding the non-significant variable, led to the following refined surface model:

(4) $${\mathrm{IC}}_{50}=49.97+1.16\times 10^{-1}X_{1}+8.69\times 10^{-2}X_{2}-3.00\times 10^{-4}X_{1}^{2}-1.20\times 10^{-4}X_{2}^{2}-\;2.90\times 10^{-2}X_{3}^{2}+1.00\times 10^{-4}X_{1}X_{2}+2.09\times 10^{-3}X_{1}X_{3}$$

**Figure 2:**
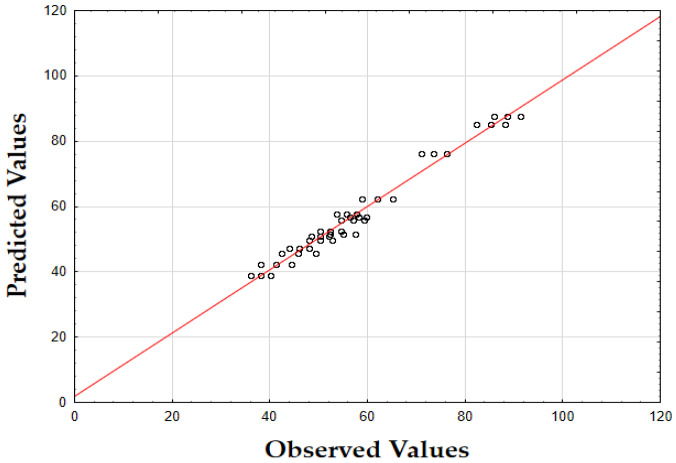
Plots of predicted versus actual values for the IC50 (mg extract/g raw material) of UAE of oregano extracts.

The F-value of 622.79 and the *p*-value of 0.00 indicate high significance in predicting the antioxidant activity. The *R*^2^ value of 0.88 confirms that the model explains most of the variability in the data. Finally, as depicted in Figure 2, the data points are closely concentrated around the diagonal, indicating very little discrepancy between the predicted and observed IC_50_ values. This tight grouping suggests minimal errors across the full range of values, confirming that the model consistently and accurately predicted the antioxidant activity of oregano extracts.

In conclusion, microwave power (*X*_1_) played a critical role in reducing IC_50_ values, indicating improved antioxidant activity in oregano extracts. This was most evident from the quadratic term for microwave power (*X*_1_^2^), which had a substantial F-value, highlighting its non-linear impact. Ultrasound power (*X*_2_) was also a key factor, particularly in its quadratic form (*X*_2_^2^), showing a significant contribution to the model’s accuracy in predicting IC_50_. While extraction time (*X*_3_) was less impactful in its linear form, its quadratic effect was significant, enhancing antioxidant capacity over longer durations. These findings align with other studies, such as research on *Schisandra chinensis*, where a synergistic ultrasound-microwave-assisted extraction significantly improved antioxidant activity and lowered IC_50_ values [58]. Additionally, a study on *Salix alba* found that microwave-assisted extraction produced high phenolic content and the lowest IC_50_ values, further emphasizing the efficiency of microwave power in boosting antioxidant activity [59]. This research underscores the importance of optimizing both microwave and ultrasound power to enhance the antioxidant properties of oregano extracts.

##### TPC

The response surface model for total phenolic content (TPC) demonstrated an excellent fit with an *R*^2^ value of 0.91 (Table 1). The quadratic model for TPC is expressed as:

TPC = 39.09 + 1.57 × 10^−^^1^ *X*_1_ − 1.90 × 10^−4^
*X*_1_^2^ + 4.89 × 10^−^^2^ *X*_2_ − 4.00 × 10^−^^5^ *X*_2_^2^ − 6.34 *X*_3_ + 4.00 × 10^−^^1^ *X*_3_^2^ − 4.00 × 10^−^^5^ *X*_1_*X*_2_ − 4.07 × 10^−^^3^ *X*_1_*X*_3_ + 9.40 × 10^−^^4^ *X*_2_*X*_3_ (5)

The quadratic term for microwave power (*X*_1_^2^) had the greatest influence on TPC, with an F-value of 95.11 (*p* < 0.001), indicating a non-linear effect of microwave power on phenolic content extraction. The linear term for ultrasound power (*X*_2_) also played a significant role (F = 119.99, *p* < 0.001), showing that increasing ultrasound power significantly enhances phenolic extraction. The quadratic term for ultrasound power (*X*_2_^2^) was also significant (F = 14.67, *p* < 0.001), suggesting a non-linear relationship between ultrasound power and TPC. However, the linear term for extraction time (*X*_3_) was not significant (F = 1.90, *p* > 0.05), showing that extraction time did not have a linear effect on TPC. Additionally, the interaction between microwave power and ultrasound power (*X*_1_*X*_3_) was moderately significant (F = 28.29, *p* < 0.001). A new regression analysis resulted in the following refined model:

(6) $$TPC=13.52+1.51\times 10^{-1}X_{1}+4.95\times 10^{-2}X_{2}-1.80\times 10^{-4}X_{1}^{2}-3.00\times 10^{-5}\;X_{2}^{2}\;+5.75\times 10^{-2}X_{3}^{2}-4.00\times 10^{-5}X_{1}X_{2}-4.39\times 10^{-3}X_{1}X_{3}$$

**Figure 3:**
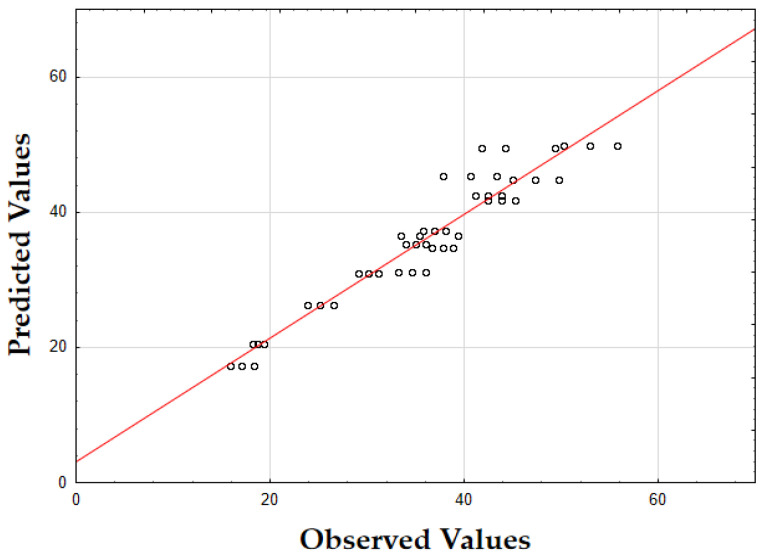
Plots of predicted versus actual values for the TPC (mg GAE/g raw material) of oregano extracts.

The F-value of 532.74 and the *p*-value of 0.00 indicate high significance in predicting TPC. The *R*^2^ value of 0.88 confirms that the model explains a substantial portion of the variability. As shown in Figure 3, the predicted values closely match the actual values, with data points tightly clustered along the diagonal. This close alignment indicates minimal deviations between the observed and predicted TPC values [60]. The small errors across the data range suggest that the model effectively captures the factors affecting the total phenolic content in oregano extracts.

In conclusion, microwave power (*X*_1_) emerged as the most influential factor affecting the total phenolic content (TPC) in oregano extracts, particularly through its quadratic term (*X*_1_^2^), which had the highest F-value, indicating a strong non-linear effect. Ultrasound power (*X*_2_) also contributed significantly, both in its linear and quadratic terms, enhancing TPC extraction. The interaction between microwave power and ultrasound power (*X*_1_*X*_2_) further improved the model’s accuracy, demonstrating that the combined effects of these variables play a critical role in maximizing phenolic content. These findings align with previous studies on phenolic compound extraction. For instance, a study on *Origanum vulgare* showed the significant effect of ultrasound-assisted extraction in maximizing phenolic compound recovery, where the optimal extraction conditions were determined using the response surface methodology [61]. Similarly, research on cotton-lavender (*Santolina chamaecyparissus* L.) demonstrated that microwave-assisted extraction outperformed ultrasound methods in extracting phenolic compounds with higher yields under optimized conditions [47]. This study underscores the importance of finetuning microwave and ultrasound power settings to achieve optimal TPC extraction in oregano.

#### 2.1.2. Interpretation of the Response Surface Model and Contour Plot

The response surface plots demonstrate the effects of microwave (MW) power, ultrasound (US) power, and extraction time on the extraction yield, antioxidant activity (IC_50_), and total phenolic content (TPC) of oregano ethanolic extracts.

**Figure 4:**
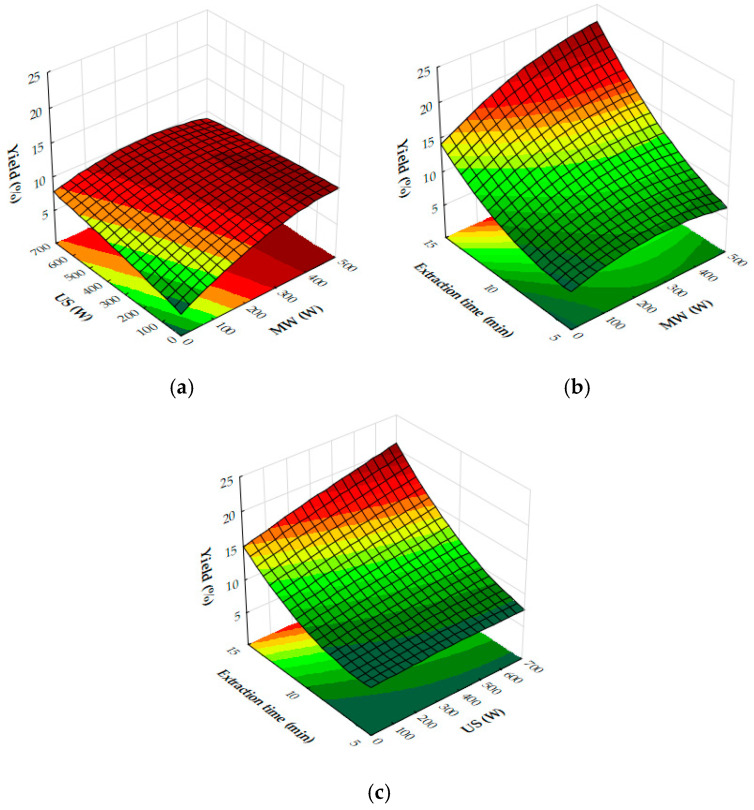
Response surface and contour plots showing the effects of MW power, US power, extraction time on the extraction yield (%) of oregano extracts. (**a**) MW power vs. US power (extraction time: 8 min); (**b**) MW power vs. extraction time (US power: 450 W); (**c**) US power vs. extraction time (MW power: 200 W).

**Figure 5:**
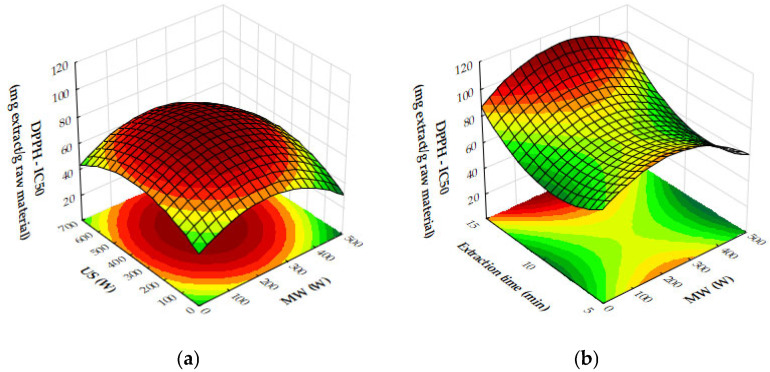
Response surface and contour plots showing the effects of MW power, US power, extraction time on IC_50_ (mg extract/g raw material) values of oregano extracts. (**a**) MW power vs. US power (extraction time: 8 min); (**b**) MW power vs. extraction time (US power: 450 W).

**Figure 6:**
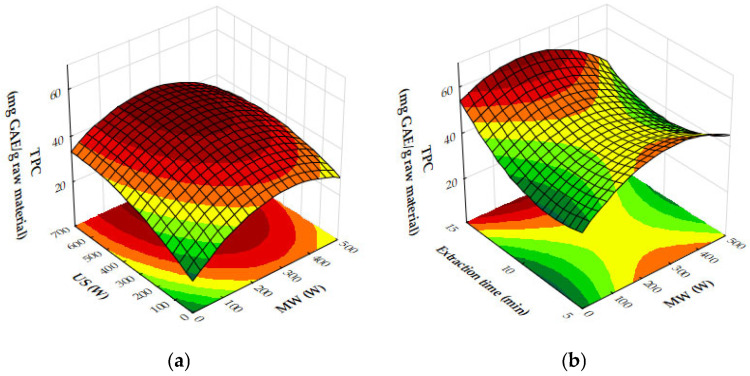
Response surface and contour plots showing the effects of MW power, US power, extraction time on TPC (mg GAE/g raw material) values of oregano extracts. (**a**) MW power vs. US power (extraction time: 8 min); (**b**) MW power vs. extraction time (US power: 450 W).

Figure 4a–c show that both MW and US power have a positive impact on the extraction yield. MW power, particularly in the range of 400–500 W, significantly increases yield, especially when paired with moderate to high US power (450 to 700 W) or extraction times of 10 to 12 min. Extraction time plays a crucial role, with the most notable improvements in yield occurring within the first 10 to 12 min, after which the yield plateaus. Beyond these levels of power or time, further improvements are minimal, suggesting diminishing returns. Figure 5a,b reveal that antioxidant activity, as measured by IC_50_ values, improves as MW and US power increase. The optimal conditions for minimizing IC_50_ values, and thus maximizing antioxidant activity, are 300 to 500 W MW power combined with 500 to 700 W US power or an extraction time of 10 to 12 min. Beyond these power levels and extraction times, the IC_50_ values plateau, indicating no significant gains in antioxidant performance. Figure 6a,b show that both MW and US power positively influence TPC, with the greatest improvements seen when 300 to 500 W MW power is applied, particularly when combined with 500 to 700 W US power or extraction times of 10 to 12 min. Extending extraction time beyond 12 min or increasing MW power above 400 W provides minimal additional benefits, signaling diminishing returns.

From the plots, the best extraction conditions for oregano ethanolic extracts are 400 to 500 W MW power, 500 to 700 W US power, and an extraction time of 10 to 12 min. These settings balance the extraction yield, antioxidant activity, and phenolic content efficiently, without requiring excessive energy input or time, while avoiding diminishing returns.

The results of the response surface plots align well with the optimal experimental results obtained for oregano ethanolic extracts. The plots indicate that both microwave (MW) power and ultrasound (US) power have a significant positive impact on the extraction yield, with the highest yields observed when MW power is set at 400 to 500 W and US power is at 500 to 700 W, aligning closely with the optimal experimental condition of 500 W MW power and 700 W US power. Additionally, the plots for DPPH IC_50_ values show that antioxidant activity improves at these power levels, supporting the experimental result of strong DPPH scavenging activity (50.31 mg extract/g). Similarly, the total phenolic content (TPC) increases significantly under these conditions, with the optimal experimental result (34.99 mg GAE/g) falling within the range of values predicted by the response surface plot. Furthermore, extraction times of 10 to 12 min are confirmed by the plots as ideal for maximizing yield, TPC, and antioxidant activity, consistent with the optimal condition of 12 min in the experimental results. Thus, the response surface plots corroborate the experimentally determined best conditions for oregano ethanolic extraction.

### 2.2. Recovery of Rosemary Extracts

The recovery of ethanolic extracts from rosemary (*Rosmarinus officinalis*) was assessed under various ultrasound and microwave power levels, combined with different extraction times. Ultrasound power ranged from 450 W to 700 W, and microwave power from 200 W to 500 W, with extraction times between 5 and 12 min. The best overall extraction conditions for rosemary ethanolic extract were achieved with 200 W microwave power and 700 W ultrasound power for 8 min, resulting in the highest yield (23.36%). This condition also provided strong DPPH scavenging activity (40.75 mg extract/g) and a notable total phenolic content of 26.35 mg GAE/g.

#### 2.2.1. Model Fitting

The table below presents the analysis of variance (ANOVA) results for the response surface models, evaluating the effects of various factors on the extraction yield (*Y*%), IC_50_ (mg extract/g raw material), and total phenolic content (TPC) (mg GAE/g raw material) in rosemary extracts.

##### Extraction Yield

The results from fitting the experimental data to the response surface model for the extraction yield (*Y*, %) of rosemary extracts are summarized in Table 2. Τhe model demonstrated a very good fit, as shown by the coefficient of determination (*R*^2^) of 0.92, indicating that 92% of the variability in the extraction yield could be explained by the model. The significance of each term was determined by F-values and *p*-values, with higher F-values and lower *p*-values indicating the most significant factors affecting the extraction yield.

The fitted surface model for the extraction yield (Y%) is represented by the following equation:

*Υ* (%) = −16.00 + 3.29 × 10^−^^2^ *X*_1_ − 4.66 × 10^−^⁵ *X*_1_^2^ − 3.00 × 10^−^^2^ *X*_2_ + 5.14 × 10^−^^5^ *X*_2_^2^ + 6.41 × *X*_3_ − 3.39 × 10^−^^1^ *X*_3_^2^ + 2.13 × 10^−^^6^ *X*_1_*X*_2_ − 4.81 × 10^−^^4^ *X*_1_*X*_3_ + 2.69 × 10^−4^ *X*_2_*X*_3_ (7)

Among the model terms, the linear effect of ultrasound power (*X*_2_) was the most significant (F = 140.95, *p* < 0.001), showing that ultrasound power had the strongest influence on the extraction yield. This was followed by the quadratic effect of ultrasound power (*X*_2_^2^), which also had a significant impact (F = 125.96, *p* < 0.001). Extraction time (*X*_3_) was another important factor, with its linear term showing high significance (F = 70.48, *p* < 0.001) and its quadratic term also contributing notably (F = 63.29, *p* < 0.001). The linear effect of microwave power (*X*_1_) was also important, with an F-value of 36.41 (*p* < 0.001), indicating that increasing microwave power enhanced the extraction yield. Non-significant terms included the interaction effects between microwave power and ultrasound power (*X*_1_*X*_2_), which had an F-value of 0.43 (*p* > 0.05), and the interaction between microwave power and extraction time (*X*_1_*X*_3_), which was also non-significant (F = 2.15, *p* > 0.05).

These findings suggest that ultrasound power, microwave power, and extraction time, especially their linear and quadratic effects, were the most influential factors in the extraction process. Based on these results, a second run was performed, excluding the non-significant variables. The updated fitted surface model is as follows:

*Υ* (%) = −16.00 + 2.96 × 10^−^^2^ *X*_1_ − 2.73 × 10^−^^2^ *X*_2_ + 6.39 × 10^0^ *X*_3_ − 4.66 × 10^−^^5^ *X*_1_^2^ + 5.14 × 10^−^^5^ *X*_1_^2^ − 3.39 × 10^−^^1^ *X*_3_^2^ (8)

**Figure 7:**
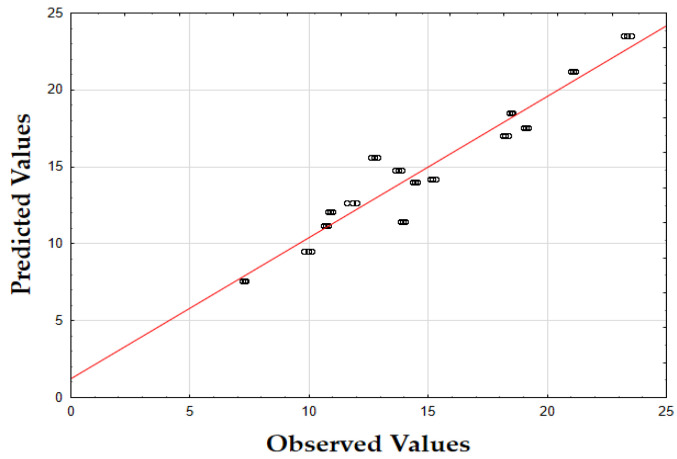
Plots of predicted versus actual values for the Y (%) of rosemary extracts.

The updated model demonstrated a good fit with an *R*^2^ value of 0.91. The data points closely align with the predicted values, confirming the model’s accuracy. Finally, as shown in Figure 7, the data points are closely grouped along the diagonal, indicating very little difference between the predicted and observed extraction yield (*Y*) values [62]. This suggests that the errors were minimal across the entire value range, demonstrating that the model reliably and accurately predicted the extraction yield of rosemary extracts.

In conclusion, ultrasound power (*X*_2_) emerged as the most significant factor affecting the extraction yield (*Y*%) of rosemary extracts, with both its linear and quadratic effects showing the highest F-values, indicating its strong influence on the process. Extraction time (*X*_3_) also played a key role, as both its linear and quadratic terms were significant contributors to the model. Additionally, the linear effect of microwave power (*X*_1_) demonstrated a notable impact on enhancing the extraction yield. However, the interaction terms between microwave power and ultrasound power (*X*_1_*X*_2_) and between microwave power and extraction time (*X*_1_*X*_3_) were non-significant, suggesting that these combined effects had little influence on the outcome. These findings are consistent with studies on the extraction of bioactive compounds from rosemary, where ultrasound- and microwave-assisted methods were shown to enhance yields, particularly when optimized for time and power settings [46]. Additionally, research on the solvent-free extraction of rosemary compounds using microwave hydrodiffusion demonstrated how optimized microwave conditions could significantly improve the recovery of essential oils and phenolic compounds [63]. This study underscores the importance of controlling extraction parameters like ultrasound power and extraction time to achieve maximum efficiency in rosemary extraction processes.

##### IC_50_

For IC_50_, representing antioxidant activity, the response surface model demonstrated an excellent fit (Table 2), with an *R*^2^ value of 0.97, as seen in the first run. The mathematical model for IC_50_ is expressed by the following equation:

IC_50_ = 58.80 − 2.40 × 10^−^^2^ X_1_ + 1.95 × 10^−^^5^ X_1_^2^ − 5.31 × 10^−^^3^ X_2_ − 3.61 × 10^−^^5^ X_2_^2^ − 6.10 × X_3_ + 3.22 × 10^−^^1^ X_3_^2^ + 1.31 × 10^−^^4^ X_1_X_2_ + 1.70 × 10^−^^3^ X_1_X_3_ + 2.66 × 10^−^^3^ X_2_X_3_ (9)

Among the independent variables, the linear term for microwave power (*X*_1_) had the most significant impact (F = 367.82, *p* < 0.001), indicating that microwave power plays a major role in reducing IC_50_ and improving antioxidant activity. Similarly, the linear term for ultrasound power (*X*_2_) also showed a strong impact (F = 209.48, *p* < 0.001), highlighting that increasing ultrasound power significantly enhances antioxidant activity.

The quadratic effect of ultrasound power (*X*_2_^2^) was another significant factor (F = 11.51, *p* < 0.01), indicating that non-linear changes in ultrasound power also contribute to antioxidant capacity. In addition, the interaction between microwave power and ultrasound power (*X*_1_*X*_2_) had a substantial influence (F = 300.16, *p* < 0.001), suggesting that these two variables work synergistically to improve antioxidant properties. The interaction between ultrasound power and extraction time (*X*_2_*X*_3_) was also significant (F = 24.03, *p* < 0.001), demonstrating that these factors combined play a role in maximizing antioxidant activity.

On the other hand, the quadratic effect of microwave power (*X*_1_^2^) was not significant (F = 0.96, *p* > 0.05), indicating that non-linear changes in microwave power do not have a notable impact on antioxidant activity. Similarly, the interaction between microwave power and extraction time (*X*_1_*X*_3_) had a lower but still significant effect (F = 4.97, *p* < 0.05), suggesting a moderate influence on the antioxidant potential of the extracts.

In summary, these findings reveal that microwave power, ultrasound power, and their interaction were the most significant factors influencing the antioxidant activity of rosemary extracts. The combined effects of these factors proved essential in enhancing antioxidant properties, as reflected in the reduction of IC_50_ values. A second run was conducted to refine the model, excluding non-significant variables to achieve a more accurate prediction of IC_50_. The refined model is expressed as:

IC_50_ = 60.20 − 1.40 × 10^−^^2^ *X*_1_ − 7.34 × 10^−^^3^ *X*_2_ − 6.56 × *X*_3_ − 3.31 × 10^−^^5^ *X*_2_^2^ + 3.49 × 10^−^^1^ *X*_3_^2^ + 1.31 × 10^−^^4^ *X*_1_*X*_2_ + 1.69 × 10^−^^3^ *X*_1_*X*_3_ + 2.65 × 10^−^^3^ *X*_2_*X*_3_ (10)

The refined model demonstrated an F-value of 940.24 (*p* < 0.001), indicating that it was highly significant in predicting the antioxidant activity of rosemary. The *R*^2^ value of 0.96 confirms that the model accounted for most of the variability in the data, providing a reliable prediction of IC_50_ under the tested conditions.

**Figure 8:**
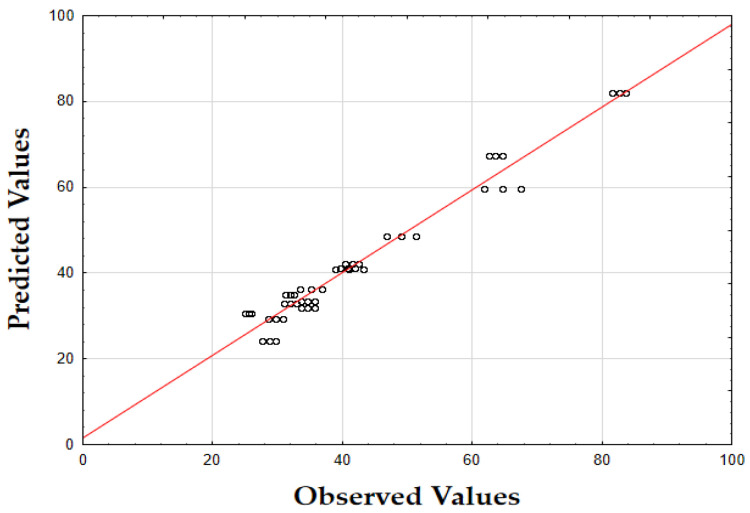
Plots of predicted versus actual values for the IC_50_ (mg extract/g raw material) of UAE of rosemary extracts.

As shown in the corresponding Figure 8, the predicted IC_50_ values align closely with the actual values, with data points tightly clustered along the diagonal line [64]. This close alignment suggests minimal deviation between observed and predicted values, indicating that the model performs well across the entire range of IC_50_ data points.

In conclusion, microwave power (*X*_1_) emerged as the most influential factor in reducing IC_50_, thus enhancing the antioxidant activity of rosemary extracts. Both the linear and interaction terms for microwave power showed high significance, with the linear term having the greatest impact (F = 367.82, *p* < 0.001). Ultrasound power (*X*_2_) also played a crucial role, as indicated by both its linear and quadratic effects, which significantly contributed to the reduction in IC_50_ values. Additionally, the quadratic term for extraction time (*X*_3_^2^) was found to be significant, demonstrating the positive influence of longer extraction times on antioxidant activity. The interaction between microwave power and ultrasound power (*X*_1_*X*_2_) was particularly noteworthy, further emphasizing their combined effect in optimizing antioxidant capacity.

These findings are consistent with studies on *Rosmarinus officinalis*, where microwave hydrodiffusion significantly improved antioxidant activity and essential oil quality [65], and similar results were reported in research on *Eucommia ulmoides*, where ultrasound-microwave-assisted extraction significantly reduced IC_50_ values and enhanced antioxidant capacity [66]. This study underscores the importance of optimizing both microwave and ultrasound power settings to maximize antioxidant activity in rosemary extracts.

##### TPC

For total phenolic content (TPC), the model also provided an excellent fit, with an *R*^2^ value of 0.91. The quadratic model for TPC is represented by the following equation:

TPC = 13.50 + 2.92 × 10^−^^2^ *X*_1_ − 4.05 × 10^−^^5^ *X*_1_^2^ − 2.07 × 10^−^^2^ *X*_2_ + 3.33 × 10^−^^6^ *X*_2_^2^ + 1.70 × *X*_3_ − 1.37 × 10^−^^1^ *X*_3_^2^ + 1.95 × 10^−^^5^ *X*_1_*X*_2_ − 4.92 × 10^−^^4^ *X*_1_*X*_3_ + 2.91 × 10^−^^3^ *X*_2_*X*_3_ (11)

The linear term for ultrasound power (*X*_2_) had the greatest influence on TPC, with an F-value of 125.91 (*p* < 0.001), indicating that increasing ultrasound power significantly enhances the extraction of phenolic compounds. Similarly, the linear term for microwave power (*X*_1_) showed a strong impact (F = 68.15, *p* < 0.001), confirming that microwave power is also a key factor in maximizing phenolic content extraction. The quadratic effect of microwave power (*X*_1_^2^) was significant (F = 12.21, *p* < 0.001), highlighting the non-linear relationship between microwave power and TPC extraction.

Moreover, the linear term for extraction time (*X*_3_) showed a moderate but significant effect (F = 6.93, *p* < 0.05), demonstrating that longer extraction times positively affect the phenolic content in the extract. The quadratic term for extraction time (*X*_3_^2^) was also significant (F = 5.60, *p* < 0.05), indicating a non-linear influence of extraction time on phenolic extraction efficiency.

In contrast, some terms were less impactful, such as the quadratic effect of ultrasound power (*X*_2_^2^) (F = 0.29, *p* > 0.05), which was not significant. The interactions between microwave power and ultrasound power (*X*_1_*X*_2_) (F = 19.39, *p* < 0.001) and between ultrasound power and extraction time (*X*_2_*X*_3_) (F = 84.19, *p* < 0.001) were both significant, suggesting that the combined effects of these variables further enhanced phenolic extraction. However, the interaction between microwave power and extraction time (*X*_1_*X*_3_) was not significant (*p* > 0.05), showing minimal combined influence on TPC.

These findings highlight the significant roles of ultrasound power, microwave power, and extraction time in optimizing the phenolic content of rosemary extracts. The linear and quadratic effects of both ultrasound power and microwave power are crucial, while the interactions between variables also play a role in fine-tuning the extraction process. A second run was conducted to refine the model by excluding non-significant variables. The updated fitted surface model for TPC is expressed by the following equation:

(12) $$TPC=15.02+2.42\times 10^{-2}X_{1}-1.85\times 10^{-2}X_{2}+1.42\times X_{3}-4.00\times 10^{-5}X_{1}^{2}-1.28\times 10^{-1}X_{3}^{2}\;+2.00\times 10^{-5}X_{1}X_{2}+2.91\times 10^{-3}X_{2}X_{3}\;$$

The updated model showed an F-value of 845.07 (*p* < 0.001), indicating a strong statistical significance in predicting total phenolic content. The *R*^2^ value of 0.90 demonstrates that the model explains most of the variability in the data, making it a reliable predictor of TPC in rosemary extracts.

**Figure 9:**
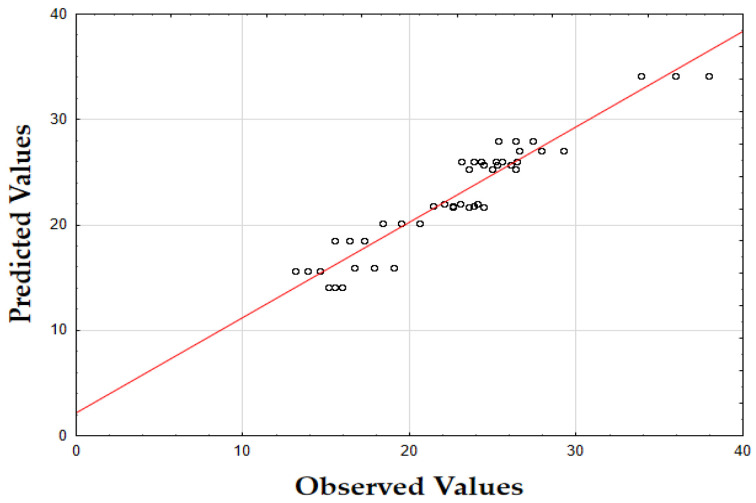
Plots of predicted versus actual values for the TPC (mg GAE/g raw material) of rosemary extracts.

As shown in Figure 9, the predicted values align closely with the actual values, with data points tightly clustered along the diagonal. This close alignment indicates minimal deviations between the observed and predicted TPC values, supporting the model’s accuracy in predicting the phenolic content across the range of data points [64].

In conclusion, ultrasound power (*X*_2_) had the most significant impact on TPC, as demonstrated by its high F-values and low *p*-values in both the linear and interaction terms. This finding suggests that maximizing ultrasound power is essential for improving the phenolic content extraction in rosemary. Microwave power (*X*_1_) also played a critical role, contributing significantly to TPC, especially in its linear form. The quadratic terms for both microwave power and extraction time (*X*_3_^2^) further emphasized the importance of non-linear effects in the phenolic extraction process. The interactions between ultrasound power and extraction time (*X*_2_*X*_3_) were also significant, indicating the combined influence of these factors in enhancing TPC extraction.

These results align with previous studies that have emphasized the importance of optimizing extraction parameters for phenolic compounds in medicinal plants. For example, research on *Satureja macrostema* found that microwave-ultrasound-assisted extraction significantly increased TPC and antioxidant activity, supporting the critical role of these techniques in phenolic recovery [67]. Additionally, recent research on *Scenedesmus obliquus* demonstrated the superior phenolic and carotenoid yields obtained using microwave-assisted extraction, further emphasizing the importance of optimizing microwave power for bioactive compound recovery [48]. These findings reaffirm the critical role of ultrasound- and microwave-assisted extraction in maximizing phenolic content across a range of plant materials.

#### 2.2.2. Interpretation of the Response Surface Model and Contour Plot

The response surface plots demonstrate how microwave (MW) power, ultrasound (US) power, and extraction time influence the DPPH IC_50_ values and total phenolic content (TPC) of rosemary ethanolic extracts.

**Figure 10:**
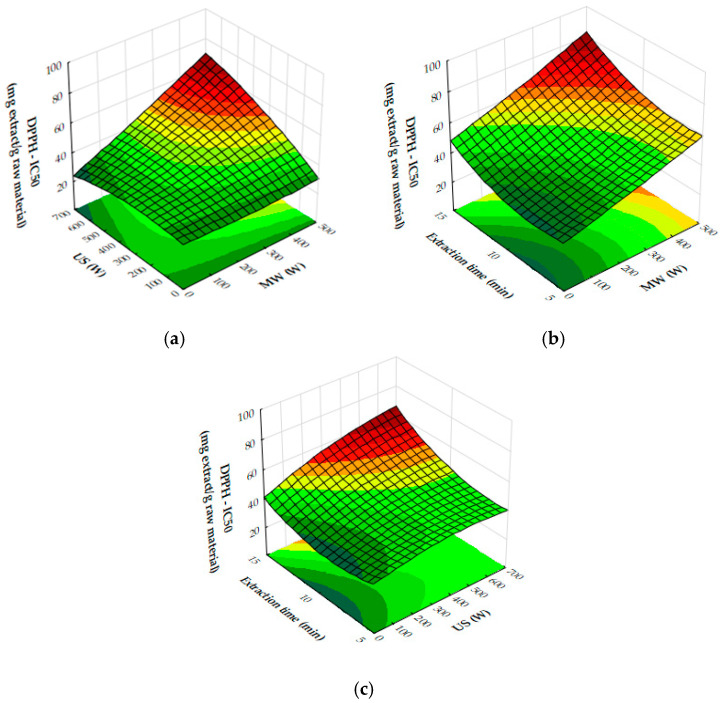
Response surface and contour plots showing the effects of MW power, US power, extraction time on IC_50_ (mg extract/g raw material) values of rosemary extracts. (**a**) MW power vs. US power (extraction time: 8 min); (**b**) MW power vs. extraction time (US power: 450 W); (**c**) US power vs. extraction time (MW power: 200 W).

**Figure 11:**
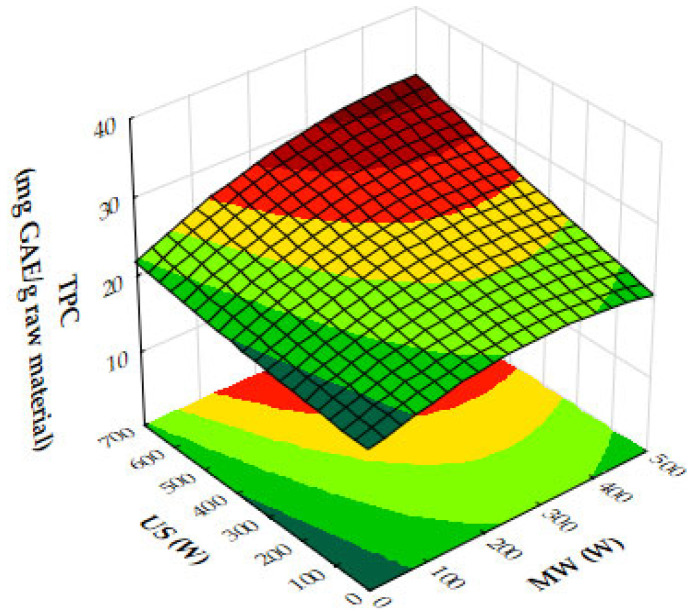
Response surface and contour plots showing the effects of MW power and US power on TPC (mg GAE/g raw material) values of rosemary extracts.

In Figure 10a, the plot demonstrates that increasing both microwave (MW) and ultrasound (US) power significantly enhances antioxidant activity, as seen through decreasing IC_50_ values. The strongest antioxidant activity is observed when MW power is in the range of 400–500 W and US power is between 600 and 700 W. Beyond these power levels, further increases lead to diminishing returns, indicating that the maximum antioxidant capacity has been reached under these conditions. Figure 10b illustrates the influence of MW power and extraction time on IC_50_ values. As MW power increases to 300 to 500 W and extraction time extends to 10 to 12 min, the IC_50_ values decrease, reflecting an improvement in antioxidant activity. However, once these thresholds are exceeded, the reduction in IC_50_ values plateaus, suggesting limited additional gains in antioxidant performance. In Figure 10c, the combined effects of US power and extraction time similarly show that stronger antioxidant activity is achieved with US power levels ranging from 400 to 700 W and extraction times of 10 to 12 min. Beyond these ranges, further increases in US power or extraction time result in diminishing returns, with no significant improvement in antioxidant activity. Finally, Figure 11 demonstrates the impact of both MW and US power on total phenolic content (TPC). The plot indicates that the most effective phenolic recovery occurs when MW power is set between 300 and 500 W and US power is in the range of 500–700 W. Beyond these optimal levels, further increases in either MW or US power provide minimal additional benefit, as the TPC values begin to plateau.

From the plots, the optimal conditions for maximizing rosemary ethanolic extract yield, TPC, and antioxidant activity (lower IC_50_) are expected to be 400 to 500 W MW power, 500 to 700 W US power, and an extraction time of 10 to 12 min. These conditions offer the best balance for extraction efficiency, phenolic content recovery, and antioxidant performance, while avoiding diminishing returns at higher power or longer times.

The results from the response surface plots align well with the optimum experimental conditions for the recovery of rosemary ethanolic extracts. The plots indicate that both microwave (MW) and ultrasound (US) power, along with extraction time, significantly influence antioxidant activity (IC_50_), total phenolic content (TPC), and yield. The experimental results identified 200 W MW power, 700 W US power, and an extraction time of 8 min as the optimal conditions, yielding 23.36% extraction, strong DPPH scavenging activity (40.75 mg extract/g), and a notable TPC of 26.35 mg GAE/g. These conditions fall within the ranges suggested by the plots, which also indicate that moderate MW power (200 W) and high US power (600 to 700 W) result in strong antioxidant activity and high phenolic content. Furthermore, the extraction time of 8 min aligns with the trend shown in the plots, where optimal results for yield, IC_50_, and TPC occur within 10 to 12 min. Thus, the experimental conditions are consistent with the trends observed in the plots, confirming that 200 W MW power, 700 W US power, and 8 min of extraction provide an efficient balance between power input and extraction effectiveness.

### 2.3. Recovery of Hypericum Extracts

The recovery of ethanolic extracts from hypericum (*Hypericum perforatum*) was evaluated under varying conditions of microwave (MW) power, ultrasound (US) power, and extraction time. MW power ranged from 0 to 500 W, US power ranged from 0 to 700 W, and extraction times were between 5 and 12 min. The optimum conditions for a achieving high extraction yield, low IC_50_ (indicating strong antioxidant activity), and high total phenolic content (TPC) were identified as 200 W MW power, 450 W US power, and an extraction time of 12 min. Under these conditions, the extraction yield reached 14.49%, with an IC_50_ value of 29.78 mg extract/g, and a TPC of 53.67 mg GAE/g. These results suggest that a balanced combination of moderate MW and US power over a slightly extended extraction time maximized both the recovery of bioactive compounds and the antioxidant capacity of the extracts.

#### 2.3.1. Model Fitting

The table below presents the analysis of variance (ANOVA) results for the response surface models, evaluating the effects of various factors on the extraction yield (Y%), IC_50_ (mg extract/g raw material), and total phenolic content (TPC) (mg GAE/g raw material) in hypericum extracts.

##### Extraction Yield

The results of fitting the experimental data to the response surface model for the extraction yield (*Y*, %) of hypericum extracts are summarized in Table 3. The model demonstrated an excellent fit to the data, as indicated by a coefficient of determination (*R*^2^) of 0.94. This value suggests that 94% of the variation in the extraction yield could be explained by the model. The statistical significance of each term was evaluated using F-values and *p*-values, where higher F-values in relation to smaller *p*-values highlight the most impactful factors on the yield.

The fitted surface model for the extraction yield (*Y*, %) is represented by the following equation:

*Υ* (%) = 6.60 + 4.31 *X*_1_ − 4.65 *X*_1_^2^ + 4.98 *X*_2_ + 5.39 *X*_2_^2^ − 6.16 *X*_3_ + 4.08 *X*_3_^2^ − 2.22 *X*_1_*X*_2_ + 4.62 *X*_1_*X*_3_ + 4.11 *X*_2_*X*_3_ (13)

Among the model terms, the linear effect of microwave power (*X*_1_) was the most significant (F = 404.40, *p* < 0.001), demonstrating that this parameter had the greatest influence on the extraction process. Following this, the quadratic term for microwave power (*X*_1_^2^) also exhibited a significant non-linear impact on yield (F = 50.59, *p* < 0.001). The linear term for ultrasound power (*X*_2_) was another significant factor (F = 32.49, *p* < 0.001), indicating the importance of ultrasound power in enhancing the extraction. Additionally, the interaction between microwave and ultrasound power (*X*_1_*X*_2_) proved to be highly significant (F = 79.20, *p* < 0.001), suggesting a strong combined effect of these two variables on the extraction yield. The linear effect of extraction time (*X*_3_) also had a moderate influence (F = 11.76, *p* < 0.01), highlighting that the duration of the extraction plays a role in the process.

In contrast, the quadratic term for ultrasound power (*X*_2_^2^) was not significant (*p* > 0.05), indicating that non-linear variations in ultrasound power did not have a substantial effect on yield. Similarly, the interactions between microwave power and extraction time (*X*_1_*X*_3_) and between ultrasound power and extraction time (*X*_2_*X*_3_) were found to be non-significant (*p* > 0.05), suggesting minimal combined effects between these variables on the yield.

These findings indicate that microwave power, ultrasound power, their interaction, and extraction time were significant factors in the extraction process. As a result, a new regression analysis was performed, omitting the non-significant variables from the extraction yield (*Y*) model. The updated fitted surface model is represented by the following equation:

*Υ* (%) = 2.98 + 4.34 × 10^−2^ *X*_1_ + 8.63 × 10^−3^ *X*_2_ + 1.97 × 10^−1^ *X*_3_ − 3.94 × 10^−5^ *X*_1_^2^ − 2.23 × 10^−5^ *X*_1_*X*_2_ (14)

**Figure 12:**
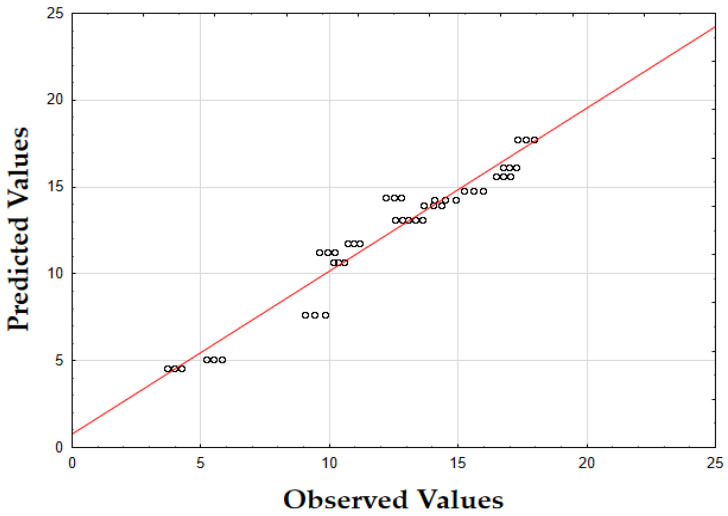
Plots of predicted versus actual values for the *Y* (%) of hypericum extracts.

The model’s F-value of 934.29 and a *p*-value of 0.00 indicate high significance in predicting the extraction yield of hypericum extracts. The high value of *R*^2^ (0.93) demonstrates that the model accounts for most of the variability in the data, providing a robust fit. Finally, as illustrated in Figure 12, the data points are densely clustered along the diagonal, indicating minimal deviation between the predicted and observed values for the extraction yield (*Y*) [64]. This implies that the errors across the entire range of values were small, demonstrating that the model consistently and accurately predicted the extraction yield of hypericum extracts.

In conclusion, microwave power (*X*_1_) emerged as the most impactful variable on the extraction yield (*Y*) of hypericum, as evidenced by its substantial F-values and low *p*-values in both the linear and quadratic forms. Ultrasound power (*X*_2_) also played a key role, particularly in its interaction with microwave power (*X*_1_*X*_2_), which significantly influenced the extraction yield. These results align with other studies focusing on the extraction of bioactive compounds from phytogenic raw materials [31]. For instance, the optimization of microwave-assisted extraction (MAE) of bioactive compounds from *Orthosiphon stamineus* identified microwave power as a critical parameter for maximizing yield, showing consistent extraction results even when scaled up [68]. Additionally, ultrasound power was highlighted in the ultrasound-microwave-assisted extraction of *Andrographis paniculata*, significantly improving the extraction yield when combined with microwave power [69]. This study confirms the necessity of fine-tuning these parameters to achieve optimal extraction yields for hypericum extracts.

##### IC_50_

For IC_50_, the experimental data demonstrated an excellent fit to the quadratic model, with an *R*^2^ value of 0.92, as seen in Table 3. The mathematical model for IC_50_ is expressed by the following equation:

IC_50_ = 5.84 + 1.56 × 10^−2^
*X*_1_ + 3.39 × 10^−4^
*X*_1_^2^ − 1.47 × 10^−1^
*X*_2_ + 2.54 × 10^−4^
*X*_2_^2^ − 1.78 *X*_3_ + 4.45 × 10^−1^
*X*_3_^2^ − 1.88 × 10^−4^
*X*_1_*X*_2_ − 9.48 × 10^−3^
*X*_1_*X*_3_ − 4.67 × 10^−3^
*X*_2_*X*_3_ (15)

Among the independent variables, the linear term for ultrasound power (*X*_2_) had a major impact on IC_50_ values (F = 84.84, *p* < 0.001), indicating that increasing ultrasound power significantly reduces IC_50_, which is associated with higher antioxidant activity. The quadratic term for ultrasound power (*X*_2_^2^) was also highly significant (F = 46.06, *p* < 0.001), illustrating the non-linear relationship between ultrasound power and IC_50_, further enhancing the extract’s antioxidant capacity.

The quadratic term for microwave power (*X*_1_^2^) also showed a significant influence (F = 23.54, *p* < 0.001), indicating a non-linear effect of microwave power on IC_50_ and thus on antioxidant activity. Moreover, the interaction between microwave power and ultrasound power (*X*_1_*X*_2_) was highly significant (F = 49.39, *p* < 0.001), suggesting that these two factors combined significantly enhance antioxidant activity. The linear term for microwave power (*X*_1_) (F = 20.99, *p* < 0.001) also contributed to the improvement of antioxidant activity by reducing IC_50_ values. Finally, the interaction between microwave power and extraction time (*X*_1_*X*_3_) was moderately significant (F = 12.47, *p* < 0.01), indicating that this interaction also affects the antioxidant properties of the extract.

The only term that was not significant in influencing IC_50_ was the quadratic term for extraction time (*X*_3_^2^) (F = 1.63, *p* > 0.05), suggesting that non-linear changes in extraction time did not significantly affect the antioxidant activity of the extract.

These results demonstrate that ultrasound power (*X*_2_), microwave power (*X*_1_), and their interaction play a crucial role in enhancing the antioxidant activity of hypericum extracts, as evidenced by the reduction in IC_50_ values. In contrast, the quadratic term for extraction time (*X*_3_^2^) did not significantly affect antioxidant activity. A new regression analysis was conducted, excluding the non-significant variable, resulting in a refined prediction of IC_50_.

The revised fitted surface model is expressed by the following equation:

IC_50_ = 3.04 × 10^1^ + 3.33 × 10^−3^ *X*_1_ − 1.56 × 10^−1^ *X*_2_ + 5.84 *X*_3_ + 3.64 × 10^−4^ *X*_1_^2^ + 2.68 × 10^−4^ *X*_2_^2^ − 1.88 × 10^−4^ *X*_1_*X*_2_ − 9.50 × 10^−3^ *X*_1_*X*_3_ − 4.65 × 10^−3^ *X*_2_*X*_3_ (16)

The model’s F-value of 202.59 and a *p*-value of 0.00 indicate high significance in predicting the antioxidant activity of hypericum extracts, as measured by IC_50_ values. The strong *R*^2^ value of 0.91 demonstrates that the model explains the majority of the variability in the data, providing a robust fit for the experimental results.

**Figure 13:**
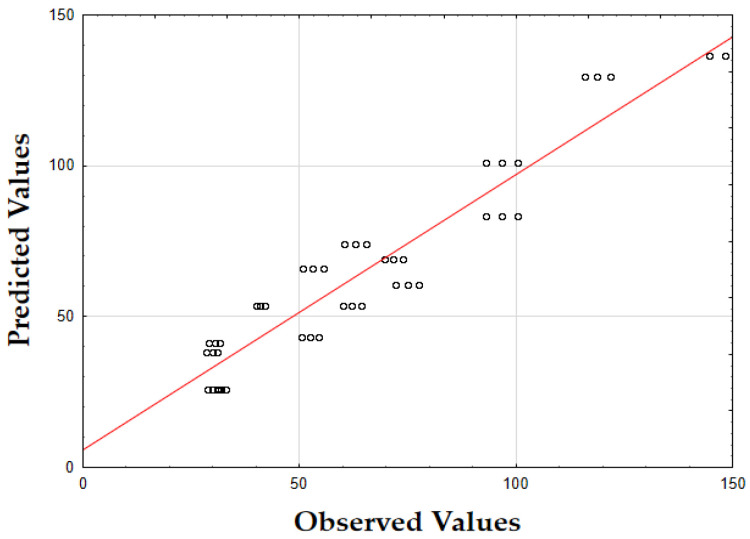
Plots of predicted versus actual values for the IC_50_ (mg extract/g raw material) of UAE of hypericum extracts.

Finally, as shown in Figure 13, the data points are densely concentrated around the diagonal, indicating minimal deviation between the predicted and observed values for IC_50_. This tight clustering implies small errors across the entire range of values, demonstrating that the model consistently and accurately predicted the antioxidant activity of hypericum extracts.

In conclusion, ultrasound power (*X*_2_) emerged as the most influential factor on the antioxidant activity of the extracts, as reflected by its significant impact in both the linear and quadratic terms. microwave power (*X*_1_) also played a key role, particularly in its interaction with ultrasound power (*X*_1_*X*_2_), which significantly enhanced antioxidant activity. These results align with similar studies on the extraction of bioactive compounds from medicinal plants. For example, the optimization of ultrasound-assisted extraction of bioactive compounds from *Eucommia ulmoides* leaves emphasized the critical role of ultrasound power in enhancing both extraction efficiency and antioxidant activity [66]. Moreover, the combination of microwave and ultrasound power was found to improve the extraction yield and antioxidant properties of bioactive compounds from brown macroalgae, highlighting the potential synergistic effects of these technologies [70]. This study confirms the importance of fine-tuning these parameters to optimize the antioxidant activity of plant extracts.

##### TPC

For TPC, the model fit was excellent, with an *R*^2^ value of 0.94, as shown in Table 3. The quadratic model for TPC is expressed by the following equation:

TPC = −9.94 + 5.15 × 10^−2^
*X*_1_ − 6.31 × 10^−5^
*X*_1_^2^ + 7.39 × 10^−2^
*X*_2_ − 8.32 × 10^−5^
*X*_2_^2^ + 8.26 *X*_3_ − 4.16 × 10^−1^
*X*_3_^2^ + 1.30 × 10^−5^
*X*_1_*X*_2_ − 7.00 × 10^−4^
*X*_1_*X*_3_ − 1.95 × 10^−4^
*X*_2_*X*_3_ (17)

Among the independent variables, the linear term for ultrasound power (*X*_2_) had the most significant impact on TPC values (F = 144.76, *p* < 0.001), showing that ultrasound power greatly enhances the extraction of phenolic compounds. The quadratic term for ultrasound power (*X*_2_^2^) also had a major influence (F = 87.93, *p* < 0.001), highlighting the non-linear relationship between ultrasound power and phenolic content extraction. The linear term for microwave power (*X*_1_) (F = 85.35, *p* < 0.001) was another critical factor in improving the TPC, indicating that microwave power substantially contributes to the extraction efficiency. The quadratic term for microwave power (*X*_1_^2^) was also significant (F = 14.56, *p* < 0.001), indicating that there is a non-linear influence of microwave power on the extraction of phenolic compounds. Additionally, the linear term for extraction time (*X*_3_) had a significant effect on TPC values (F = 42.86, *p* < 0.001), showing that extending the extraction duration enhances the total phenolic content. In contrast, some terms were less influential or non-significant. The interaction between microwave power and ultrasound power (*X*_1_*X*_2_) (F = 4.23, *p* < 0.05) had a minor significant effect. However, the interactions between microwave power and extraction time (*X*_1_*X*_3_) (*p* > 0.05) and ultrasound power and extraction time (*X*_2_*X*_3_) (*p* > 0.05) were not significant, indicating minimal combined effects on TPC.

These results indicate that ultrasound power (*X*_2_), microwave power (*X*_1_), and extraction time (*X*_3_) play critical roles in enhancing the total phenolic content of hypericum extracts. The quadratic effects of both ultrasound power and microwave power further influence the TPC, demonstrating that fine-tuning these parameters is essential for maximizing the extraction of phenolic compounds. A new regression analysis was conducted, excluding non-significant variables, resulting in a refined prediction of TPC. The revised fitted surface model is expressed as follows:

TPC = −7.91 × 10^0^ + 4.55 × 10^−2^ *X*_1_ + 7.23 × 10^−2^ *X*_2_ + 8.01 *X*_3_ + 6.30 × 10^5^ *X*_1_^2^ + 8.33 × 10^−5^ *X*_2_^2^ − 4.16 × 10^−1^ *X*_3_^2^ + 1.30 × 10^−5^ *X*_1_*X*_2_ (18)

The model’s F-value of 1335.95 and a *p*-value of 0.00 indicate that the model is highly significant in predicting the total phenolic content (TPC) of hypericum extracts. The high *R*^2^ value of 0.94 confirms that the model explains most of the variability in the data, making it a reliable fit for predicting TPC under the tested conditions.

**Figure 14:**
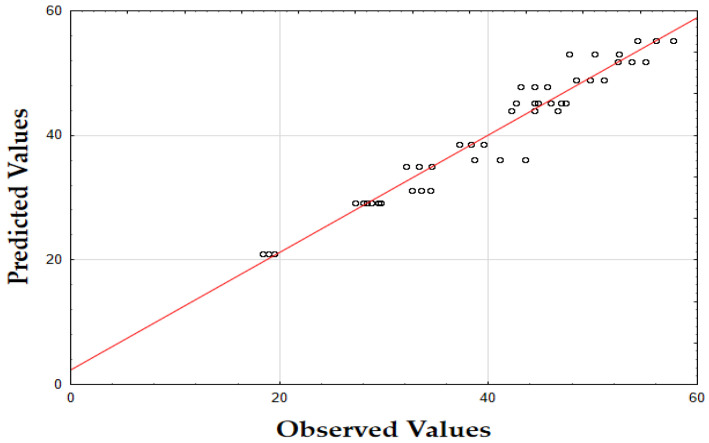
Plots of predicted versus actual values for the TPC (mg GAE/g raw material) of hypericum extracts.

As illustrated in Figure 14, the predicted values align closely with the actual values, with data points densely clustered along the diagonal line. This tight clustering indicates minimal deviations between the observed and predicted TPC values [64]. Such small errors across the range of data imply that the model accurately captures the factors influencing the total phenolic content in hypericum extracts.

In conclusion, ultrasound power (*X*_2_) had the most substantial impact on TPC, as shown by its high F-values and low *p*-values in both the linear and quadratic terms. This suggests that increasing ultrasound power is critical for maximizing the extraction of phenolic compounds. Microwave power (*X*_1_) also played a crucial role, contributing significantly to TPC, especially in its linear form. The extraction time (*X*_3_) also emerged as an important factor, with a strong influence on the phenolic content when applied for extended durations. However, the interactions between microwave power and extraction time (*X*_1_*X*_3_) and ultrasound power and extraction time (*X*_2_*X*_3_) were not significant, indicating that these factors do not interact in a way that notably affects phenolic extraction. These findings align with previous research on phenolic compound extraction from other medicinal plants. For example, Sarakatsianos et al. (2020) optimized microwave-assisted extraction of phenolic compounds from *Sideritis raeseri* and *Origanum vulgare*, identifying microwave power as a crucial variable for enhancing phenolic yield [71]. Similarly, Kaderides et al. (2019) demonstrated that microwave power significantly improved the yield of phenolics from pomegranate peels, while ultrasound power further enhanced the extraction process [50].

#### 2.3.2. Interpretation of the Response Surface Model and Contour Plot

**Figure 15:**
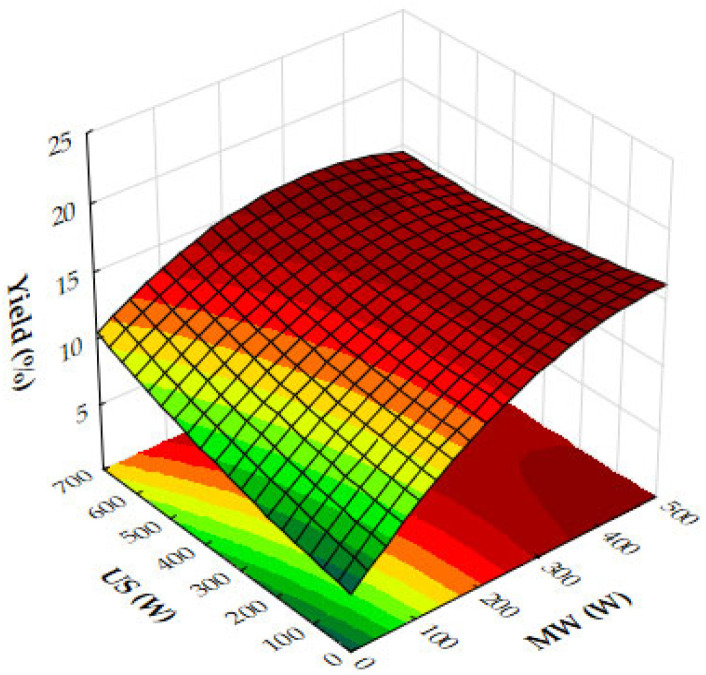
Response surface and contour plots showing the effects of MW power and US power on the extraction yield (%) of hypericum extracts.

The response surface plot (Figure 15) demonstrates the combined effects of microwave (MW) and ultrasound (US) power on the extraction yield of hypericum ethanolic extracts. MW power (0–500 W) and US power (0–700 W) both positively influence yield, with MW having a stronger effect at moderate US power levels. Yield increases with both variables but plateaus at high US power, indicating diminishing returns. Optimal conditions for maximizing yield involve moderate to high MW power combined with moderate US power, as excessive US power provides little additional benefit.

**Figure 16:**
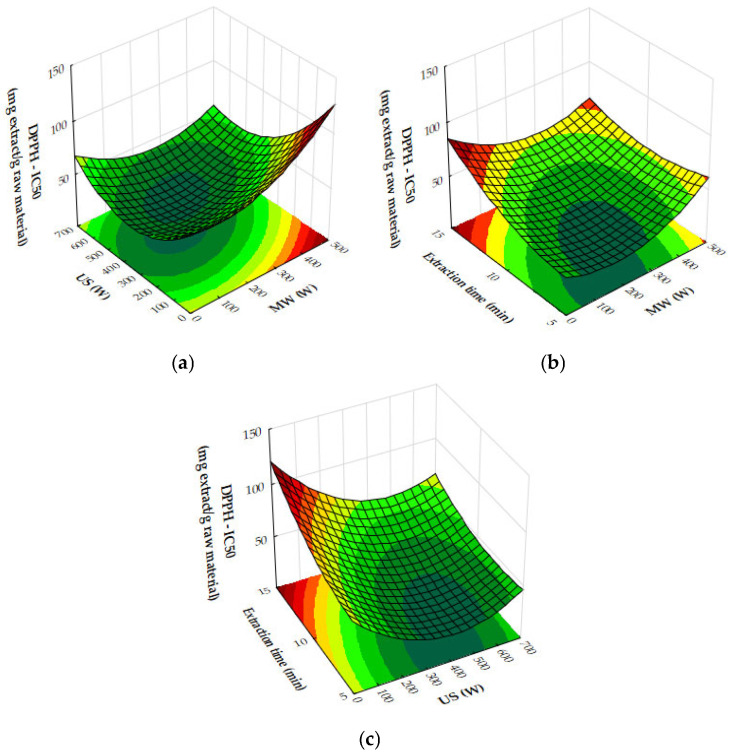
Response surface and contour plots showing the effects of MW power, US power, extraction time on IC_50_ (mg extract/g raw material) values of hypericum extracts. (**a**) MW power vs. US power (extraction time: 8 min); (**b**) MW power vs. extraction time (US power: 450 W); (**c**) US power vs. extraction time (MW power: 200 W).

In Figure 16a, the combined effects of MW and US power on DPPH IC_50_ values (antioxidant activity) are illustrated with a fixed extraction time of 8 min. Antioxidant activity improves as MW and US power increase to moderate levels, with the lowest IC_50_ values occurring when both are applied in balance. Beyond a certain threshold, higher power levels result in reduced antioxidant activity, as reflected by increasing IC_50_ values. The optimal conditions for antioxidant activity are achieved with moderate MW and US power, beyond which performance declines.

Figure 16b highlights the effects of MW power and extraction time on DPPH IC_50_ values, with US power held constant at 450 W. Antioxidant activity improves as MW power and extraction time increase to moderate levels, but beyond approximately 300 W and 10 min, IC_50_ values rise, indicating a decline in antioxidant efficacy. The plot suggests that the optimal conditions for antioxidant recovery involve moderate MW power and extraction times, while excessive power or prolonged extraction reduces antioxidant activity.

Figure 16c shows the effects of US power and extraction time on DPPH IC_50_ values with MW power held constant at 200 W. Antioxidant activity improves with US power up to around 450 W and extraction time up to 10 min, beyond which IC_50_ values plateau or rise. The optimal antioxidant activity occurs at moderate US power and extraction times, while excessive levels lead to reduced performance.

**Figure 17:**
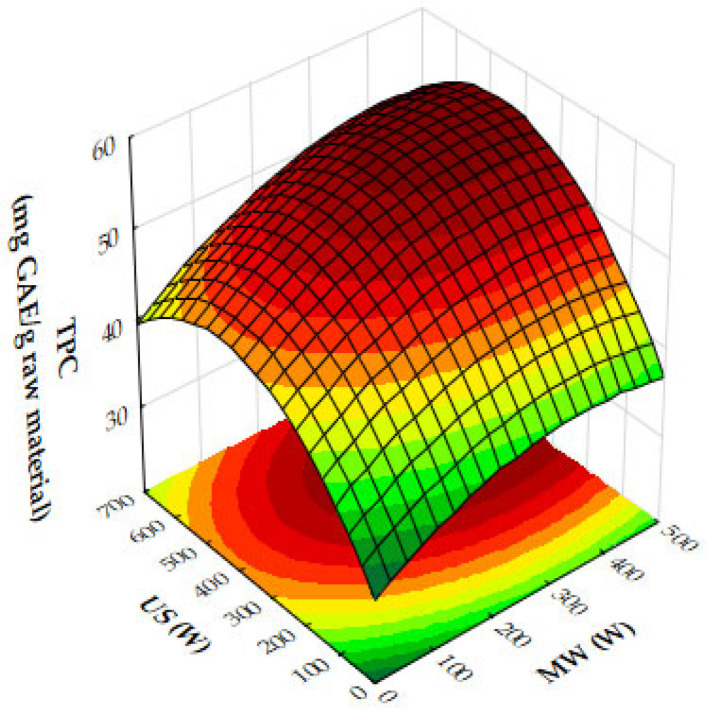
Response surface and contour plots showing the effects of MW power and US power on TPC (mg GAE/g raw material) values of hypericum extracts.

Finally, Figure 17 presents the effects of MW and US power on total phenolic content (TPC). Both MW and US power enhance phenolic recovery, with MW power exerting a stronger influence. At moderate US power levels (around 450 W), TPC increases significantly. However, at high MW power, further increases in US power provide diminishing returns. The optimal phenolic recovery is achieved with high MW power and moderate to high US power, which together maximize bioactive compound extraction.

Taking into account the response surface and contour plots for total phenolic content (TPC), DPPH antioxidant activity, and yield, the optimal extraction conditions appear to involve a combination of both moderate to high microwave (MW) power and ultrasound (US) power, and an extraction time of 10–12 min. This combination offers a well-balanced outcome across all three parameters: high extraction yield, strong antioxidant activity (as reflected by low DPPH IC_50_ values), and elevated total phenolic content.

The results align with the experimentally determined optimal conditions of 200 W microwave power, 450 W ultrasound power, and 12 min extraction time, which were previously determined for the recovery of ethanolic extracts from hypericum dried leaves. This consistency across both datasets reinforces the effectiveness of moderate microwave and ultrasound power settings in maximizing the extraction of bioactive compounds while maintaining the integrity and functionality of the extracts. While higher microwave power (e.g., 500 W) can boost yield, the plots indicate that extending the extraction time to 12 min at moderate microwave and ultrasound power levels maximizes both the bioactivity and phenolic recovery, making it the most effective overall condition for obtaining a high-quality extract. This balanced approach ensures that all critical factors—yield, bioactivity, and phenolic content—are optimized simultaneously, leading to a superior extract.

### 2.4. Recovery of Chamomile Extracts

The recovery of ethanolic extracts from chamomile (*Matricaria recutita*) was optimized by evaluating different conditions of microwave (MW) power, ultrasound (US) power, and extraction time. MW power ranged from 0 to 500 W, US power from 0 to 700 W, and extraction times from 5 to 12 min. The optimal conditions for achieving a balanced extract, with high yield, strong antioxidant activity (low IC_50_), and elevated total phenolic content (TPC), were found to be 500 W MW power, 700 W US power, and an extraction time of 5 min. Under these conditions, the extraction yield was 17.79%, the IC_50_ value was 55.82 mg extract/g, and the TPC was 12.85 mg GAE/g. These results provide an ideal balance, maximizing both the recovery of bioactive compounds and the antioxidant potential of the extracts.

#### 2.4.1. Model Fitting

The table below presents the analysis of variance (ANOVA) results for the response surface models, evaluating the effects of various factors on the extraction yield (Y%), IC_50_ (mg extract/g raw material), and total phenolic content (TPC) (mg GAE/g raw material) in chamomile extracts.

##### Extraction Yield

The response surface model was used to analyze the effect of microwave (MW) power, ultrasound (US) power, and extraction time on the extraction yield (*Y*, %) of chamomile extracts. As summarized in Table 4, the model demonstrated an excellent fit, with an *R*^2^ value of 0.93, suggesting that 93% of the variability in the extraction yield could be explained by the model. The statistical significance of the terms was evaluated using F-values and *p*-values, where higher F-values and smaller *p*-values indicated the most impactful factors on the yield.

The quadratic model for the extraction yield (*Y*) is represented by the following equation:

*Υ* (%) = 148.00 + 2.85 × 10^−^^2^
*X*_1_ − 2.17 × 10^−^^5^
*X*_1_^2^ + 1.14 × 10^−^^2^
*X*_2_ + 3.01 × 10^−^^6^
*X*_2_^2^ − 3.04 × 10^0^
*X*_3_ + 2.14 × 10^−^^1^
*X*_3_^2^ − 2.46 × 10^−^^5^
*X*_1_*X*_2_ + 3.03 × 10^−^^4^
*X*_1_*X*_3_ + 2.41 × 10^−^^4^
*X*_2_*X*_3_ (19)

Among the significant terms, the linear effect of ultrasound power (*X*_2_) was found to have the greatest influence on the extraction yield (F = 196.54, *p* < 0.001). This indicates that ultrasound power plays the most crucial role in maximizing the yield. Following this, the linear effect of microwave power (*X*_1_) also exhibited a strong influence (F = 153.25, *p* < 0.001), demonstrating that microwave power is another critical factor in the extraction process. The linear term for extraction time (*X*_3_) also showed substantial significance (F = 128.73, *p* < 0.001), emphasizing the importance of extraction duration.

Additionally, the interaction between microwave and ultrasound power (*X*_1_*X*_2_) was significant (F = 69.22, *p* < 0.001), indicating a strong combined effect of these two variables on the extraction yield. The quadratic term for extraction time (*X*_3_^2^) also demonstrated significance (F = 30.91, *p* < 0.001), highlighting that non-linear changes in extraction time further enhance the yield. In contrast, the quadratic effect of microwave power (*X*_1_^2^) had a smaller but still significant effect (F = 7.87, *p* < 0.01).

However, the quadratic term for ultrasound power (*X*_2_^2^) was not significant (F = 0.53, *p* > 0.05), indicating that non-linear changes in ultrasound power do not substantially impact the yield. Similarly, the interaction between microwave power and extraction time (*X*_1_*X*_3_) and ultrasound power and extraction time (*X*_2_*X*_3_) were not significant (F = 1.04 and 1.30, respectively, *p* > 0.05).

In summary, the linear effects of ultrasound power, microwave power, extraction time, and their interaction (*X*_1_*X*_2_) were the most significant factors affecting the extraction yield. Based on these results, a new regression analysis was conducted, excluding non-significant variables, to refine the model for Y%. The refined fitted surface model is expressed as follows:

*Υ* (%) = 13.90 + 3.03 × 10^−^^2^ *X*_1_ + 1.54 × 10^−^^2^ *X*_2_ − 3.02 × 10^0^ *X*_3_ − 2.02 × 10^−^^5^ *X*_1_^2^ + 2.23 × 10^−^¹ *X*_3_^2^ − 2.46 × 10^−^^5^ *X*_1_*X*_2_ (20)

**Figure 18:**
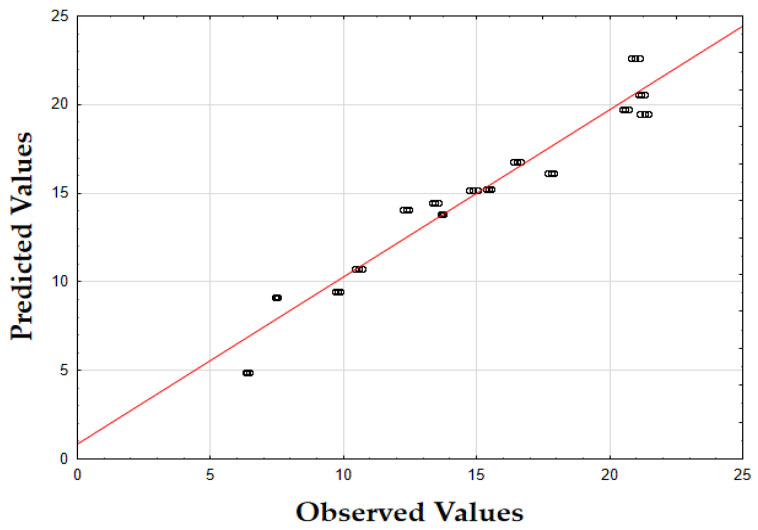
Plots of predicted versus actual values for the *Y* (%) of chamomile extracts.

The model’s F-value of 947.61 and a *p*-value of 0.00 indicate a strong significance in predicting the extraction yield for chamomile. The coefficient of determination (*R*^2^) of 0.94 shows that the model explains most of the variability in the data, indicating an excellent fit. As shown in Figure 18, the predicted versus actual values for *Y* (%) are tightly clustered along the diagonal, confirming a minimal deviation between predicted and observed values and demonstrating the model’s accuracy.

In conclusion, ultrasound power (*X*_2_) emerged as the most impactful variable on the extraction yield (*Y*) of chamomile, as indicated by its high F-value and low *p*-value in the linear term. This suggests that increasing ultrasound power significantly improves the yield of chamomile extracts. Microwave power (*X*_1_) was also a key factor, showing strong influence in both its linear and quadratic forms. Additionally, extraction time (*X*_3_), both in its linear and quadratic terms, played a critical role in enhancing the extraction yield. The interaction between microwave and ultrasound power (*X*_1_*X*_2_) further influenced the yield, demonstrating the importance of optimizing these parameters simultaneously for better results. These findings are consistent with other research on medicinal plants, where ultrasound- and microwave-assisted extraction were shown to increase bioactive compound yields, such as in the case of *Orthosiphon stamineus* and *elder* (*Sambucus nigra*) bark [68,72]. Optimization of ultrasound power has also been critical in improving the extraction of phenolic compounds, as demonstrated in studies on *olive leaves* and *Humulus lupulus* (hops), where microwave- and ultrasound-assisted methods dramatically enhanced extraction yields and antioxidant activity [29,73]. These results highlight the importance of fine-tuning both ultrasound and microwave parameters to achieve optimal extraction yields across various plant materials.

##### IC_50_

For IC_50_, which represents antioxidant activity (lower IC_50_ values indicate stronger antioxidant capacity), the model fitting showed an excellent fit, with an *R*^2^ value of 0.98 (Table 4). The mathematical model for IC_50_ is expressed as:

IC_50_ = 2.41 × 10^2^ − 2.39 × 10^−^^1^
*X*_1_ + 6.14 × 10^−^^5^
*X*_1_^2^ − 3.67 × 10^−^^1^
*X*_2_ + 2.50 × 10^−^^4^
*X*_2_^2^ − 1.65 × 10^1^
*X*_3_ + 1.08 × 10^0^
*X*_3_^2^ + 2.35 × 10^−^^4^
*X*_1_*X*_2_ + 1.36 × 10^−^^2^
*X*_1_*X*_3_ − 1.38 × 10^−^^3^
*X*_2_*X*_3_ (21)

The linear term for ultrasound power (*X*_2_) had the most significant influence on IC_50_ (F = 1333.72, *p* < 0.001), indicating that increasing ultrasound power greatly reduces the IC_50_ value and enhances antioxidant activity. The linear term for extraction time (*X*_3_) also had a significant effect (F = 141.61, *p* < 0.001), showing that prolonged extraction enhances antioxidant capacity.

The quadratic term for ultrasound power (*X*_2_^2^) was highly significant as well (F = 102.82, *p* < 0.001), further indicating the importance of ultrasound power for antioxidant activity. The interaction between microwave and ultrasound power (*X*_1_*X*_2_) showed substantial significance (F = 178.42, *p* < 0.001), suggesting a synergistic effect in improving antioxidant properties. Similarly, the interaction between microwave power and extraction time (*X*_1_*X*_3_) also significantly influenced IC_50_ (F = 59.68, *p* < 0.001).

However, the linear term for microwave power (*X*_1_) had a much smaller influence (F = 3.41, *p* > 0.05), and the quadratic term for microwave power (*X*_1_^2^) was also non-significant (F = 1.78, *p* > 0.05). The interaction between ultrasound power and extraction time (*X*_2_*X*_3_) was not significant (F = 1.21, *p* > 0.05), indicating minimal combined effects on antioxidant activity.

These results demonstrate that ultrasound power (*X*_2_), microwave power (*X*_1_), and their interaction play a crucial role in enhancing the antioxidant activity of chamomile extracts, as evidenced by the reduction in IC_50_ values. In contrast, the quadratic term for extraction time (*X*_3_^2^) did not significantly affect antioxidant activity. A new regression analysis was conducted, excluding the non-significant variable, resulting in a refined prediction of IC_50_.

The revised fitted surface model is expressed by the following equation:

IC_50_ = 197 − 3.66 × 10^−^^1^ *X*_2_ − 1.31 × 10^1^ *X*_3_ + 2.59 × 10^−^^4^
*X*_2_^2^ + 1.11 × 10^0^ *X*_3_^2^ + 1.53 × 10^−^^4^ *X*_1_*X*_2_ − 4.89 × 10^−^^3^ *X*_1_*X*_3_ (22)

**Figure 19:**
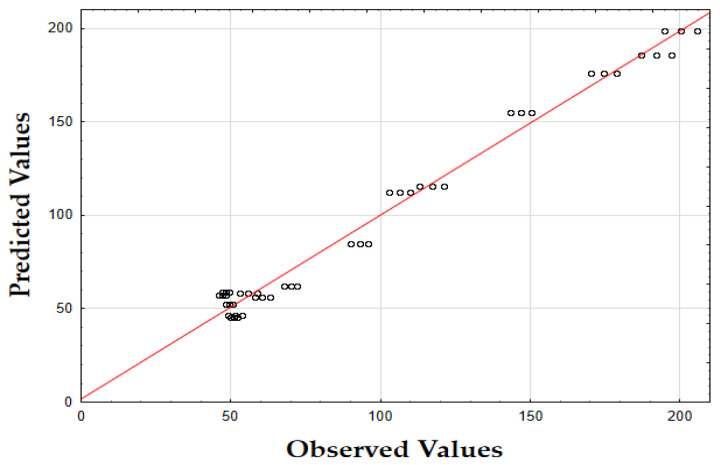
Plots of predicted versus actual values for the IC_50_ (mg extract/g raw material) of UAE of chamomile extracts.

The F-value of 278.44 and the *p*-value of 0.00 suggest that the model is highly significant in predicting antioxidant activity, as measured by IC_50_. The *R*^2^ value of 0.92 demonstrates that the model accounts for most of the variability in the data, providing a robust fit. The plot of predicted versus actual IC_50_ values (Figure 19) reveals a close clustering along the diagonal, implying that the model performs well in predicting IC_50_ values across the range of data points.

In summary, ultrasound power (*X*_2_) had the most significant influence on antioxidant activity, as demonstrated by its high F-value in both linear and quadratic terms. This indicates that increasing ultrasound power is crucial for lowering IC_50_ values and boosting the antioxidant properties of chamomile extracts. Extraction time (*X*_3_) also had a notable effect, contributing to the model in both linear and quadratic forms. Additionally, the interactions between microwave power and ultrasound power (*X*_1_*X*_2_), as well as microwave power and extraction time (*X*_1_*X*_3_), significantly enhanced antioxidant capacity. These results align with studies on other medicinal plants, such as *Eucommia ulmoides* leaves, where ultrasound-microwave-assisted extraction significantly improved antioxidant activity with reduced IC_50_ values [66]. Similarly, *Opuntia ficus indica* extracts showed enhanced antioxidant activities under increased microwave power, demonstrating that microwave power plays a crucial role in reducing IC_50_ values [74]. The combination of ultrasound and microwave extraction techniques has also been shown to improve both antioxidant and antibacterial activities in other plant-based extracts, as seen in *Schisandra chinensis* [58]. These findings reinforce the importance of optimizing ultrasound and microwave parameters to achieve maximum antioxidant activity in plant extracts.

##### TPC

For TPC, the model fit was strong, with an *R*^2^ value of 0.91, as shown in Table 4. The quadratic model for TPC is expressed by the following equation:

TPC = 4.02 × 10^0^ + 6.18 × 10^−^^3^*X*_1_ + 2.24 × 10^−^^5^*X*_1_^2^ + 2.78 × 10^−^^2^*X*_2_ − 2.03 × 10^−^^5^*X*_2_^2^ − 8.13 × 10^−^^1^*X*_3_ + 3.60 × 10^−^^2^*X*_3_^2^ − 1.91 × 10^−^^5^*X*_1_*X*_2_ + 3.46 × 10^−^^4^*X*_1_*X*_3_ + 1.25 × 10^−^^4^*X*_2_*X*_3_ (23)

The linear term for ultrasound power (*X*_2_) had the greatest impact on TPC (F = 144.03, *p* < 0.001), indicating that higher ultrasound power significantly enhances the extraction of phenolic compounds. The linear term for microwave power (*X*_1_) also showed a substantial influence on TPC (F = 140.81, *p* < 0.001).

The quadratic terms for ultrasound power (*X*_2_^2^) and microwave power (*X*_1_^2^) were both significant (F = 87.93 and 14.56, respectively, *p* < 0.001), demonstrating non-linear effects of these factors on phenolic content. The linear effect of extraction time (*X*_3_) was also significant (F = 42.86, *p* < 0.001), showing that longer extraction times positively affect TPC.

However, some terms were less impactful, such as the interaction between microwave power and ultrasound power (*X*_1_*X*_2_), which was significant but had a smaller effect (F = 4.23, *p* < 0.05). The interactions between microwave power and extraction time (*X*_1_*X*_3_) as well as between ultrasound power and extraction time (*X*_2_*X*_3_) were non-significant (F = 1.21 and 0.23, respectively, *p* > 0.05), indicating minimal combined effects between these variables on total phenolic content.

In summary, the linear effect of ultrasound power (*X*_2_) was the most influential factor on TPC, followed by microwave power (*X*_1_). The quadratic terms for both microwave and ultrasound power, as well as the linear term for extraction time (*X*_3_), were also important contributors. Non-significant interactions between variables suggest that their combined effects on TPC were negligible.

Based on these findings, a new regression analysis was conducted, excluding the non-significant terms. The updated fitted surface model for TPC is expressed as:

TPC = 1.03 + 8.21 × 10^−^^3^ *X*_1_ + 2.81 × 10^−^^2^ *X*_2_ + 2.42 × 10^−^^5^ *X*_1_^2^ − 1.92 × 10^−^^5^ *X*_2_^2^ − 1.91 × 10^−^^5^ *X*_1_*X*_2_ (24)

**Figure 20:**
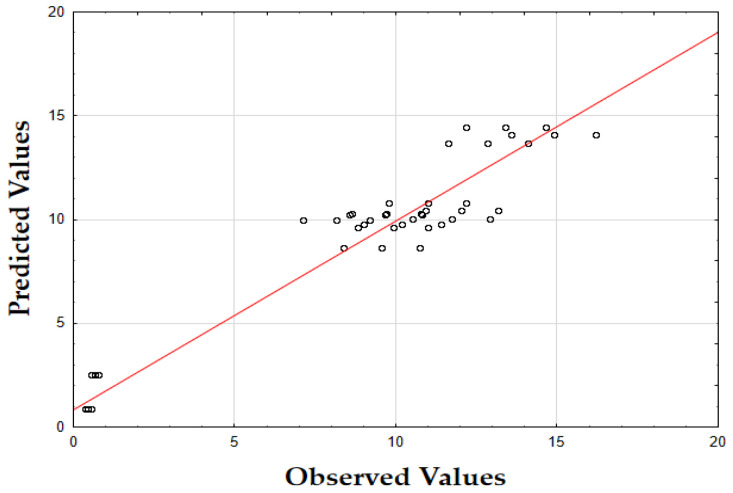
Plots of predicted versus actual values for the TPC (mg GAE/g raw material) of chamomile extracts.

The model’s F-value of 315.99 and a *p*-value of 0.00 suggest a high level of significance in predicting the total phenolic content (TPC) in chamomile extracts. With an *R*^2^ value of 0.91, the model accounts for a substantial proportion of the variability in the data, underscoring its strength. As seen in Figure 20, the close alignment of data points along the diagonal in the plot of predicted versus actual TPC values further supports the model’s predictive accuracy.

In conclusion, ultrasound power (*X*_2_) was the most influential factor affecting the total phenolic content (TPC) in chamomile extracts, as demonstrated by its strong linear and quadratic effects. Microwave power (*X*_1_) also played a significant role, particularly in its linear form, while the quadratic terms for both microwave and ultrasound power contributed to TPC. Extraction time (*X*_3_) also had an important effect, enhancing the phenolic content when extended over longer durations. Although the interactions between microwave and ultrasound power (*X*_1_*X*_2_) were less impactful, they still contributed to the overall extraction process. These results align with previous research, such as studies on *Chlorella vulgaris*, where microwave-assisted extraction significantly enhanced phenolic content and antioxidant activity compared to conventional methods [48]. Additionally, in pomegranate peels, microwave extraction was shown to increase phenolic yield and antioxidant activity, shortening the extraction process time significantly compared to ultrasound methods [50].

#### 2.4.2. Interpretation of the Response Surface Model and Contour Plot

**Figure 21:**
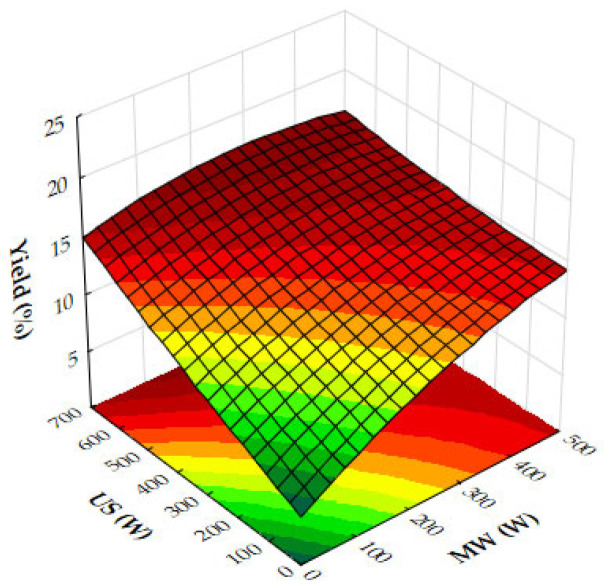
Response surface and contour plots showing the effects of MW power and US power on the extraction yield (%) of chamomile extracts.

The response surface plot of Figure 21 illustrates the combined effects of microwave (MW) power and ultrasound (US) power on the extraction yield (%) of chamomile ethanolic extracts. As MW power increases from 0 to 500 W, the yield steadily improves, particularly when US power is applied at moderate to high levels. At low MW power, increasing US power has minimal effect on the yield. The highest yields are achieved when both MW and US power are applied at higher levels, though the improvement plateaus at the upper power ranges, indicating diminishing returns. The optimal extraction yield is achieved with a combination of moderate to high MW and US power.

**Figure 22:**
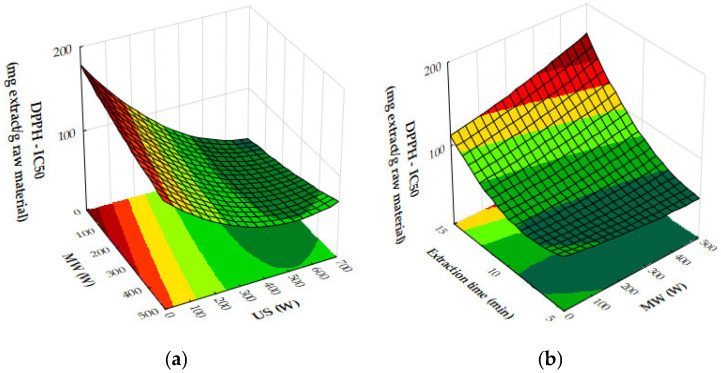
Response surface and contour plots showing the effects of MW power, US power, extraction time on IC_50_ (mg extract/g raw material) values of chamomile extracts. (**a**) MW power vs. US power (extraction time: 8 min); (**b**) MW power vs. extraction time (US power: 450 W).

Figure 22a illustrates the effects of MW and US power on DPPH IC_50_ values, which indicate antioxidant activity. Increasing both variables reduces IC_50_ values, showing enhanced antioxidant activity. The most pronounced decrease occurs with high MW power and moderate to high US power, though after a certain threshold, further increases in US power yield little additional improvement, as reflected by the flattening of the surface at the lowest IC_50_ values.

Figure 22b shows the combined effects of MW power and extraction time on IC_50_ values. As MW power and extraction time increase, IC_50_ values decrease, suggesting improved antioxidant activity. However, extraction times beyond 12 min offer little additional benefit, as IC_50_ values begin to plateau. The optimal antioxidant extraction is achieved with moderate to high MW power and extraction times around 10 to 12 min.

**Figure 23:**
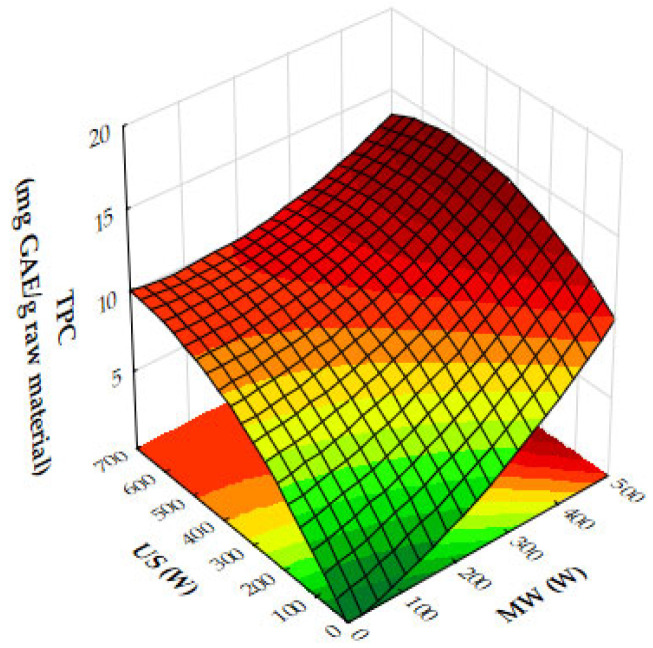
Response surface and contour plots showing the effects of MW power and US power on TPC (mg GAE/g raw material) values of chamomile extracts.

Lastly, the response surface plot of Figure 23 illustrates the combined effects of MW and US power on total phenolic content (TPC). Both variables positively affect phenolic recovery, with the most significant increases occurring at moderate to high levels of both. While MW power has a stronger effect on TPC, US power also contributes, though its benefits diminish at very high MW levels. The best results for TPC are obtained with a balanced combination of high MW and moderate to high US power.

In conclusion, the optimal conditions for chamomile ethanolic extraction, as shown in Figure 21, Figure 22 and Figure 23**,** involve a combination of moderate to high MW power, moderate US power, and an extraction time of around 10 to 12 min. These settings ensure an efficient extraction yield, strong antioxidant activity, and high phenolic content. Further increases in MW power, US power, or extraction time provide diminishing returns beyond these thresholds, making a balanced approach the most effective for maximizing extraction efficiency.

The optimal conditions for the maximizing extraction yield, total phenolic content (TPC), and reducing DPPH IC_50_ (indicating strong antioxidant activity) are expected to involve 400 to 500 W microwave (MW) power and 500 to 700 W ultrasound (US) power, combined with an extraction time of 10 to 12 min. MW power has a significant positive effect on yield and TPC, while also enhancing antioxidant activity by reducing IC_50_ values. US power further improves yield and TPC, though with diminishing returns at higher levels. The extraction time of 10 to 12 min is sufficient to optimize antioxidant performance without unnecessary prolongation, making this combination of parameters ideal for efficient bioactive compound recovery.

The response surface plot analysis aligns well with the optimal experimental conditions for chamomile ethanolic extracts, achieved at 500 W microwave (MW) power, 700 W ultrasound (US) power, and an extraction time of 5 min. The plots indicate that both MW and US power significantly enhance the extraction yield and total phenolic content (TPC), with the highest levels of both powers leading to improved recovery of bioactive compounds. The observed yield of 17.79%, an IC_50_ value of 55.82 mg extract/g (indicating strong antioxidant activity), and a TPC of 12.85 mg GAE/g closely match the predicted optimal conditions from the plots. This confirms that a combination of high MW and US power, along with a relatively short extraction time, maximizes both antioxidant potential and phenolic recovery. The balance between power input and extraction efficiency is consistent with theoretical expectations, validating the experimental results as an optimal approach for chamomile extract recovery.

### 2.5. Optimum Extracts—Comparison

The recovery of ethanolic extracts from oregano (*Origanum vulgare*), rosemary (*Rosmarinus officinalis*), hypericum (*Hypericum perforatum*), and chamomile (*Matricaria recutita*) was optimized by evaluating different conditions of microwave (MW) power, ultrasound (US) power, and extraction time. The optimal extraction conditions for each plant varied, and a comparison of their extraction yields, IC_50_ values (indicating antioxidant activity), and total phenolic content (TPC) provides insight into the effectiveness of each extraction process.

Table 5 presents the optimized ultrasound-assisted extraction (UAE) and microwave-assisted extraction (MAE) conditions for oregano, rosemary, *Hypericum perforatum*, and chamomile. The extraction parameters, including UAE power, MAE power, and extraction time, were optimized using the response surface methodology (RSM). The responses include the extraction yield (Y), total phenolic content (TPC), and antioxidant activity as measured by the IC_50_ values, highlighting the efficiency of the synergistic UAE–MAE approach.

#### 2.5.1. Oregano Ethanolic Extract

For oregano, the optimal conditions were 500 W MW power, 700 W US power, and 12 min extraction time, resulting in a yield of 16.57%. The IC_50_ value was 50.31 mg extract/g, indicating strong antioxidant activity, and the TPC reached 34.99 mg GAE/g. The higher power levels required for oregano extraction resulted in good antioxidant activity and TPC, though the yield was slightly lower than that of chamomile. The robust antioxidant activity and moderate yield reflect the specific bioactive matrix of oregano, which often requires higher energy input to achieve optimal phenolic recovery.

The obtained yield (16.57%) is consistent with results reported in other studies [30,61,75,76,77,78,79,80,81]. For example, Michalaki et al. (2023) [61] used UAE with response surface methodology (RSM) optimization to extract oregano and achieved yields of approximately 16.2% under similar ethanol concentration and power conditions. Similarly, Zeković et al. (2017) [51] reported oregano yields ranging from 15% to 18% when using UAE and MAE, depending on ethanol ratios and extraction times. These findings confirm that the yield achieved in this study is competitive with existing UAE and MAE approaches, particularly given the hybrid extraction setup.

The TPC of 34.99 mg GAE/g is also in line with literature values for oregano. Michalaki et al. (2023) [61] observed a TPC of 362.1 ± 1.8 mg GAE/g using UAE with optimized extraction parameters, demonstrating the significant phenolic content recoverable from oregano. Castro-López et al. (2017) [82] reported a TPC of 38.5 mg GAE/g when employing MAE under high-power conditions, further validating that the TPC values from the current study fall within the upper spectrum of reported results for oregano.

Antioxidant activity, measured through the IC_50_ value, provides additional evidence of the effectiveness of the extraction protocol. The IC_50_ of 50.31 mg extract/g aligns with the findings of Zeković et al. (2017) [51], who reported antioxidant activities with IC_50_ values ranging between 45 and 55 mg extract/g for oregano extracts optimized with green extraction techniques. This robust antioxidant potential is largely attributed to key bioactive compounds such as rosmarinic acid, luteolin, and apigenin, all of which were also identified in previous studies, including Michalaki et al. (2023) [61], where these compounds were quantified through HPLC analysis.

The study’s results are further validated by findings from Rodsamran and Sothornvit (2019) [83], who compared UAE and MAE for phenolic extractions and observed that UAE consistently provided higher phenolic content and antioxidant activities due to the effective disruption of plant matrices. Similarly, Garcia-Vaquero et al. (2021) [84] demonstrated that UAE–MAE combinations for bioactive extraction from plant matrices yield higher efficiency compared to single techniques, emphasizing the potential of combined methods for maximizing phenolic recovery in oregano.

In summary, the optimized UAE–MAE extraction protocol for oregano demonstrated efficient recovery of bioactive compounds, as evidenced by the high TPC (34.99 mg GAE/g), robust antioxidant activity (IC_50_ of 50.31 mg extract/g), and competitive yield (16.57%). These results align with or exceed values reported in the literature for oregano, highlighting the efficacy of the applied hybrid extraction approach. Variations across studies are likely due to differences in plant origin, solvent composition, and experimental setups. This study underscores the potential of UAE–MAE for developing sustainable and effective extraction techniques for oregano bioactives.

#### 2.5.2. Rosemary Ethanolic Extract

Rosemary exhibited the highest extraction yield of 23.36% under conditions of 200 W MW power, 700 W US power, and an extraction time of 8 min. Its IC_50_ value of 40.75 mg extract/g indicated good antioxidant activity, though not as strong as that of hypericum or oregano. The TPC was 26.35 mg GAE/g, higher than chamomile but lower than *Hypericum perforatum* and oregano. These findings reflect a highly efficient extraction process where high MW and US power levels, coupled with a shorter extraction time, maximized bioactive compound recovery.

These results align with previous studies [46,85,86,87,88,89,90,91,92,93]. For instance, Bellumori et al. (2016) reported yields of 18.7% to 20.0% for rosemary UAE and MAE extractions using ethanol as the solvent, while Jacotet-Navarro et al. (2015) [87] reported yields ranging from 13% to 20% with UAE or MAE extractions using an ethanol–water mixture. Similarly, Irakli et al. (2023) [46] achieved phenolic yields of 25.5% for rosemary using UAE and 24% using MAE with aqueous ethanol solutions, with TPC values of 25.2 mg GAE/g under 450 W ultrasonic power, a 1:20 solid-to-liquid ratio, and a 10 min extraction time. Bunghez et al. (2015) [94] documented TPC values of 15.4 mg GAE/g for rosemary extracts, while Hosseini et al. (2018) [93] achieved a TPC of 26.6 mg GAE/g using UAE at 200 W power, a 1:20 solid-to-liquid ratio, and a 12 min extraction time. Additionally, Munekata et al. (2020) [89] reported TPC values of 20% for UAE rosemary ethanolic extracts. The extraction yield in this study (23.36%) aligns closely with Pontillo et al. (2021) [91], who reported yields between 20.8% and 24.5% depending on ethanol concentration and extraction parameters during MAE and UAE.

The IC_50_ value of 40.75 mg extract/g for rosemary indicates substantial antioxidant activity, though slightly less potent than hypericum or oregano under similar conditions. This finding is consistent with Hosseini et al. (2018) [93], who reported IC_50_ values ranging from 19.2 to 24 mg/g for rosemary MAE extracts using ethanol–water mixtures. Similarly, Dhouibi et al. (2023) [92] observed IC_50_ values between 35 and 45 mg extract/g for rosemary extracted with UAE and ethanol-based solvents. Vieira et al. (2022) [95] also highlighted the stability of bioactive compounds such as carnosic acid and rosmarinic acid during UAE, further validating the effectiveness of the UAE–MAE method in preserving antioxidant potential.

The recovery of key bioactive compounds demonstrates the efficiency of the UAE–MAE method. Bellumori et al. (2016) [85] reported that UAE and MAE individually achieved rosmarinic acid recoveries of 4–6 mg/g and carnosic acid recoveries of 13–24 mg/g from dried raw material. Similarly, Jacotet-Navarro et al. (2015) [87] reported rosmarinic acid recoveries of 0.5–2.5 mg/g with UAE or MAE. Irakli et al. (2023) [46] demonstrated that UAE could yield 12.5 mg/g of carnosic acid and 3.6 mg/g of rosmarinic acid under optimized conditions, values comparable to or slightly exceeded by the UAE–MAE protocol used in this study.

In summary, the optimized UAE–MAE extraction conditions for rosemary demonstrated effective recovery of bioactive compounds, evidenced by the high yield (23.36%), substantial antioxidant activity (IC_50_ 40.75 mg extract/g), and TPC (26.35 mg GAE/g). These results align with or exceed literature-reported values for similar methods, confirming the efficiency of this approach. Variations in reported values across studies likely stem from differences in plant material, solvent composition, and extraction conditions.

#### 2.5.3. Hypericum Ethanolic Extract

For *Hypericum perforatum*, the optimal extraction conditions were 200 W microwave (MW) power, 450 W ultrasound (US) power, and an extraction time of 12 min. Under these conditions, the process achieved an extraction yield of 14.5%, an IC_50_ value of 29.8 mg extract/g (indicating strong antioxidant activity), and a high total phenolic content (TPC) of 53.7 mg GAE/g. These results represent a well-balanced extraction process, where moderate MW and US power, combined with a longer extraction time, effectively maximized the recovery of bioactive compounds and enhanced antioxidant potential.

The yield results align well with findings from other studies, which report extraction yields ranging from 2% to 26% for similar conventional, UAE, or MAE extraction methods [96,97,98,99]. For instance, Farkas et al. (2019) observed yields between 2% and 9% using conventional extraction with methanol as the solvent [100]. Similarly, Milutinović et al. (2024) achieved yields of up to 15% through MAE extraction with an ethanol–water mixture as the solvent [55]. Kakouri et al. (2023) reported yields as high as 22% using UAE extraction with a methanol–water solvent mixture [101]. These comparisons highlight that the current extraction method is consistent with or exceeds the efficiency of previously reported techniques.

The TPC also falls within the range of reported literature values. For conventional extraction techniques, TPC has been documented to range between 15 and 54 mg GAE/g dry sample [100,102], while UAE methods have achieved values as high as 64 mg GAE/g dry sample when utilizing methanol–water mixtures as the solvent [103]. Additionally, the IC_50_ value falls on the lower end of the spectrum reported in similar studies [55,97,99,100,101,102,104,105], indicating strong antioxidant activity and highlighting the efficacy of the extraction method employed in this study.

The optimized extraction conditions for *Hypericum perforatum* demonstrated effective recovery of bioactive compounds, as evidenced by the high yield (14.5%), strong antioxidant activity (IC_50_ of 29.8 mg extract/g), and elevated total phenolic content (53.7 mg GAE/g). These results align well with or exceed those reported in the literature for similar extraction techniques, further validating the efficiency of the applied UAE/MAE hybrid method. Variations in reported yields and TPC values across studies can be attributed to differences in plant material, drying methods, and the solvents used.

#### 2.5.4. Chamomile Ethanolic Extract

In the case of chamomile, the best extraction conditions were 500 W MW power, 700 W US power, and an extraction time of 5 min, yielding 17.79%. However, the IC_50_ value of 55.82 mg extract/g indicated weaker antioxidant activity compared to hypericum, while the TPC was 12.85 mg GAE/g (equivalent to 72.23 mg GAE/g chamomile extract), lower than hypericum but reflecting a reasonably balanced extraction process in a shorter time. When compared to previous studies, our results demonstrate both efficiency and effectiveness. For instance, TPC values of 117.31 mg ECA/g extract and 123.40 mg ECA/g extract were reported for MAE and UAE, respectively, using a 70% ethanol–water mixture and 40 min extraction times [106]. The longer extraction times and ethanol–water solvent likely contributed to the higher TPC values compared to our shorter, more energy-efficient protocol. Another study employing UAE at 140 W with 50% ethanol reported a TPC of 7.19 mg GAE/g extract, significantly lower than our findings, potentially due to the lower power and solvent concentration used [107]. Furthermore, water-based extraction at 80 °C for 15 min yielded a TPC of 55.98 ± 7.33 mg GAE/g powder, demonstrating the effectiveness of ethanol in phenolic extraction compared to water alone [108]. Finally, studies utilizing UAE and MAE with 70% ethanol reported TPC values ranging from 8.6 to 9.4 mg GAE/g extract, further emphasizing the efficiency of the optimized UAE–MAE conditions in our study [109]. These comparisons highlight that our method achieves competitive TPC values while offering significant time and energy efficiency advantages, underscoring its potential as a green and effective extraction approach for chamomile.

Hypericum exhibited the best overall balance, featuring the strongest antioxidant activity (lowest IC_50_), the highest total phenolic content (TPC), and a moderate extraction yield, making it the most effective extract. Rosemary, on the other hand, had the highest extraction yield (23.36%) in the shortest time (8 min), demonstrating exceptional efficiency in terms of both time and power, although its antioxidant activity and TPC were slightly lower than those of hypericum and oregano. Using 200 W microwave power, 700 W ultrasound power, and an 8 min extraction time, rosemary was the most time and power efficient. Chamomile, while achieving a high yield in a short time, had the highest IC_50_ (55.82 mg extract/g), indicating the weakest antioxidant activity.

In conclusion, while rosemary was the most efficient in terms of yield and extraction time, hypericum emerged as the best overall extract, offering the strongest antioxidant activity and the highest TPC, with a balanced yield under moderate extraction conditions. Chamomile had the weakest antioxidant properties, though it achieved high extraction efficiency.

### 2.6. HPLC Analysis of Extracts at Optimum Conditions

Each extract obtained under the optimized conditions for oregano, rosemary, Hypericum, and chamomile was analyzed using high-performance liquid chromatography with diode array detection (HPLC–DAD). To ensure accurate identification of main phenolic compounds, authentic analytical standards were employed for cross-referencing and retention time matching, and UV absorption spectra were compared with data reported in the literature. This approach effectively supports the findings derived from the optimized extraction conditions, providing a reliable screening of key phenolic composition. It should be noted, however, that while HPLC–DAD serves as a valuable and widely accepted analytical tool, further screening could benefit from more advanced techniques, such as mass spectrometry, to achieve a more detailed and comprehensive characterization of the extract profiles.

#### 2.6.1. Oregano Ethanolic Extract

**Figure 24:**
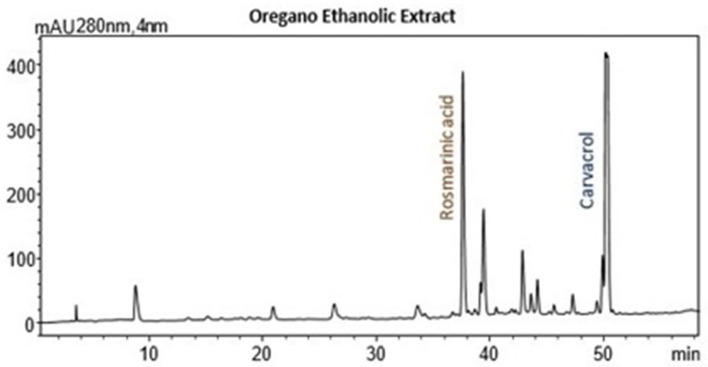
HPLC chromatograms of oregano extract recorded at 280 nm (for detection of rosmarinic acid and carvacrol).

The HPLC chromatogram of the oregano ethanolic extract (Figure 24) provides a detailed profile of its major bioactive compounds, with particular emphasis on carvacrol and rosmarinic acid. At 280 nm, the chromatogram reveals a distinct, sharp peak corresponding to carvacrol, a phenolic compound renowned for its potent antimicrobial properties. The well-defined retention time and sharp peak indicate effective separation, preservation, and efficient recovery of this compound during the extraction process. Additionally, the chromatogram highlights a prominent peak for rosmarinic acid, a phenolic acid recognized for its strong antioxidant and anti-inflammatory properties. The clear resolution of the peaks demonstrates the success of the optimized extraction process in isolating these bioactive compounds, underscoring the high-quality and bioactive-rich composition of the oregano extract.

#### 2.6.2. Rosemary Ethanolic Extract

**Figure 25:**
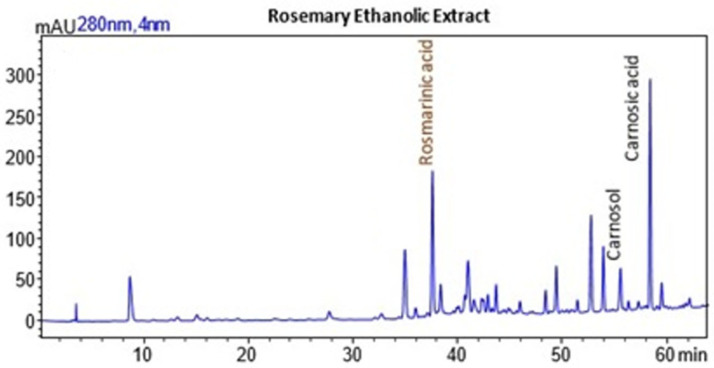
HPLC chromatograms of rosemary extract recorded at 280 nm (for detection of rosmarinic acid, carnosol, and carnosic acid).

The HPLC chromatogram of the rosemary ethanolic extract provides an analysis of its key bioactive compounds, focusing on flavonoids, rosmarinic acid, carnosic acid, and carnosol. In Figure 25 (monitored at 280 nm), distinct peaks corresponding to carnosic acid and carnosol are observed. These well-resolved peaks confirm the effective extraction of these potent antioxidant compounds, with clear retention times indicating their successful isolation and the preservation of their bioactive integrity during the extraction process. In addition, the chromatogram highlights a significant peak for rosmarinic acid. The sharp and well-defined peak indicates the substantial presence of this bioactive in the extract, emphasizing the efficacy of the optimized extraction method. Rosmarinic acid, recognized for its antioxidant and anti-inflammatory properties, follows carnosic acid in abundance. The chromatogram from Figure 25 demonstrates the rich bioactive profile of rosemary. It confirms the successful recovery and preservation of carnosic acid, carnosol, and rosmarinic acid, showcasing the quality and antioxidant potential of the rosemary extract.

#### 2.6.3. Hypericum Ethanolic Extract

**Figure 26:**
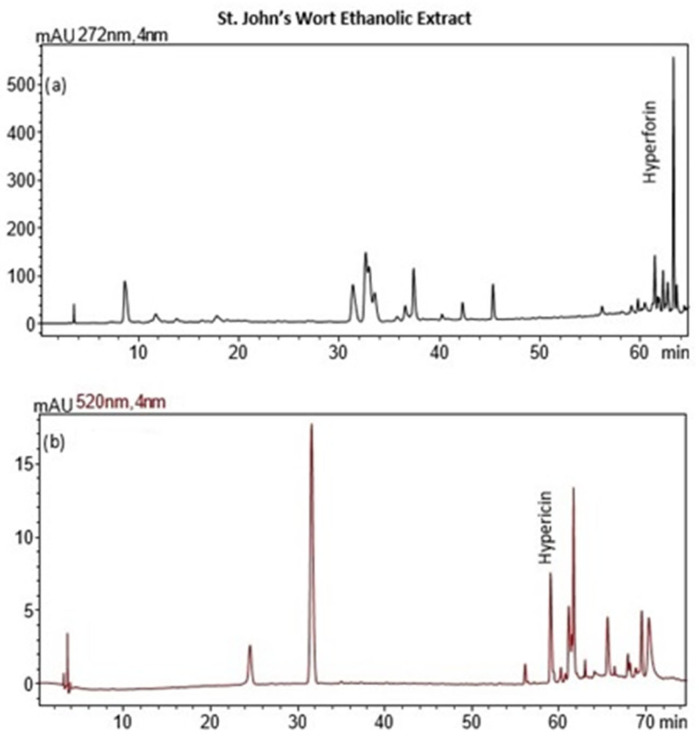
HPLC chromatograms of hypericum extract recorded at: (**a**) 272 nm (for detection of hyperforin), and (**b**) 520 nm (for detection of hypericin).

The HPLC chromatograms (Figure 26a,b) provide a detailed analysis of the key bioactive compounds extracted from Hypericum under the optimized conditions. In Figure 26a, monitored at 272 nm, a prominent peak corresponding to hyperforin is observed. Known for its antidepressant properties, hyperforin is effectively isolated with a clear retention time, reflecting the success of the extraction method. The distinct peak highlights efficient recovery and preservation of the compound’s bioactive integrity during the extraction process. In Figure 26b, monitored at 520 nm, a strong and well-resolved peak for hypericin is evident. Known for its antidepressant and antiviral properties, hypericin is successfully extracted and clearly separated from other components in the chromatogram, underscoring the effectiveness of the optimized method. Together, these chromatograms validate the optimized extraction process, demonstrating its capability to isolate and preserve hyperforin, flavonoids, and hypericin in Hypericum extracts. The results highlight a high-quality extract and a comprehensive chemical profile, suitable for further applications.

#### 2.6.4. Chamomile Ethanolic Extract

**Figure 27:**
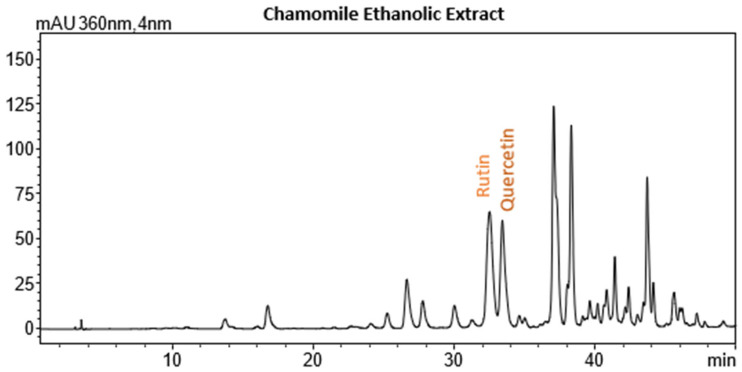
HPLC chromatogram of chamomile extract recorded at 360 nm (for detection of rutin and quercetin).

The chromatogram of the chamomile ethanolic extract (Figure 27) provides a detailed overview of the bioactive compounds detected at 360 nm, showcasing key flavonoids present in the extract. Two prominent peaks have been identified as rutin and quercetin, which are well-known flavonoids with potent antioxidant and anti-inflammatory properties. The well-resolved and sharp peaks reflect successful separation and confirm the presence of these compounds in significant concentrations, likely contributing to the antioxidant potential of the extract. This chromatogram highlights the richness of flavonoids in chamomile ethanolic extract and confirms that rutin and quercetin are among the major components contributing to its bioactive profile.

All UV spectra of the identified compounds are provided in the Supplemental Material Table S1 includes the UV spectra for the compounds identified in the oregano extract, while Table S2 presents the spectra for the rosemary extract. Additionally, Tables S3 and S4 display the UV spectra of the identified compounds in hypericum and chamomile extracts, respectively. The UV spectra of the identified compounds are consistent with those reported in the literature [104,110,111,112,113,114,115,116,117,118,119].

Table 6 provides a detailed quantification of key bioactive compounds extracted from oregano (*Origanum vulgare*), rosemary (*Rosmarinus officinalis*), *Hypericum perforatum*, and chamomile (*Matricaria recutita*) under optimized conditions utilizing ultrasound-assisted extraction (UAE) and microwave-assisted extraction (MAE).

The quantified concentrations of bioactive compounds in oregano, rosemary, *Hypericum perforatum*, and chamomile extracts, as indicated in Table 5, align well with ranges reported in the literature for ethanolic extractions, highlighting the efficacy of the optimized UAE/MAE method.

For oregano, carvacrol (10.1 ± 0.4 mg/g) and rosmarinic acid (9.2 ± 0.6 mg/g) were effectively extracted. These concentrations fall within the typical ranges reported in the literature, with carvacrol generally found between 5 and 15 mg/g and rosmarinic acid between 5 and 20 mg/g [14,120,121,122,123,124]. Previous studies, such as Oreopoulou et al. (2020), reported carvacrol at 5.5 mg/g and rosmarinic acid at 10.1 mg/g using conventional ethanol/water extraction [76], while Baranauskaitė et al. (2015) observed carvacrol levels up to 8 mg/g with ethanol/propylene glycol mixtures using UAE extraction [125]. The concentrations observed in this study reflect the optimized extraction process’s ability to enhance the recovery of these bioactives, which contribute significantly to oregano’s antimicrobial, antioxidant, and anti-inflammatory properties.

In rosemary, carnosic acid (6.6 ± 0.2 mg/g), carnosol (2.3 ± 0.2 mg/g), and rosmarinic acid (5.0 ± 0.3 mg/g) were detected at levels consistent with the literature [126,127]. Typical ranges for carnosic acid in ethanolic extracts are 5–20 mg/g, while carnosol and rosmarinic acid are usually present at 2.5–5 mg/g and up to 6 mg/g, respectively. Studies by Bellumori et al. (2016) and Jacottet-Navarro et al. (2015) reported similar concentrations using UAE and MAE methods [85,87]. The elevated levels of carnosic acid observed in this study, combined with consistent levels of carnosol and rosmarinic acid, highlight the effectiveness of the extraction method in isolating rosemary’s potent antioxidants.

For hypericum, hyperforin (10.4 ± 0.5 mg/g) and hypericin (2.1 ± 0.2 mg/g) were quantified at levels within or exceeding the ranges reported in the literature [98,101,128,129]. Hyperforin, typically reported at 3–10 mg/g, was observed at the higher end of this range, while hypericin concentrations were consistent with reported values of 1–2 mg/g. For example, Cassuta et al. (2011) documented hyperforin at 1.15 mg/g using Soxhlet extraction, highlighting the superior recovery achieved under the optimized conditions in this study [128]. These results demonstrate the protocol’s efficacy in preserving *Hypericum’s* bioactive compounds.

In chamomile, rutin (3.5 ± 0.4 mg/g) and quercetin (2.5 ± 0.3 mg/g) were detected within typical ranges for ethanolic extracts. Literature values generally report rutin between 2 and 5 mg/g and quercetin between 2 and 3 mg/g for extracts obtained through conventional, UAE, or MAE methods [106,130,131,132,133]. The levels observed in this study underscore the robustness of the extraction conditions in recovering these flavonoids, which contribute to chamomile’s antioxidant and anti-inflammatory properties.

Overall, the quantified concentrations of bioactive compounds across all extracts are consistent with or exceed the ranges reported in the literature. The findings highlight the efficiency of the synergistic UAE/MAE method in maximizing the recovery of key bioactive compounds while preserving their structural integrity. This approach underscores the potential of these extraction techniques for producing high-quality plant extracts with applications in industrial and therapeutic contexts.

## 3. Materials and Methods

### 3.1. Materials and Chemicals

Dried leaves of oregano (*Origanum vulgare*), rosemary (*Rosmarinus officinalis*), hypericum (*Hypericum perforatum*) and chamomile (*Matricaria recutita*) were purchased from a local market specializing in herbal products. The moisture content of all dried samples was measured and standardized at 8%, ensuring consistency in drying and comparability of the results. Fractions of each herb’s dried leaves with different particle sizes were first obtained through grinding using a knife mill Pulverisette 11 (Fritsch, Thessaloniki, Greece), followed by sieving with manually operated sieves of 300 μm diameter. All samples were prepared under standardized conditions to maintain uniformity and ensure the reliability of the findings. For the HPLC analysis, the chemicals and reagents used included ultrapure water, methanol, acetonitrile, and isopropanol, all of which were HPLC grade. Additionally, trifluoroacetic acid and phosphoric acid were utilized. All reagents were sourced from Sigma-Aldrich (St. Louis, MO, USA). The authentic analytical standards of rosmarinic acid, carvacrol, rutin, quercetin, hyperforin, hypericin, carnosic acid, and carnosol, each with a purity greater than 98%, were supplied by Extrasynthese S.A. (Genay Cedex, France).

### 3.2. Ultrasound-Assisted Extraction (UAE) and Microwave-Assisted Extraction (MAE)

Ultrasonic-assisted extraction (UAE) and microwave-assisted extraction (MAE) were utilized for the extraction of each herb, as well as a combination of both techniques (UAE–MAE). The extraction was performed using the XO-SM50 Ultrasonic Microwave Reaction System (Nanjing Xianou Instruments Manufacture Co., Ltd., Nanjing, China). Ethanol served as the solvent, with a solid-to-liquid ratio set at 1:20 (g/mL). Microwave power (W), ultrasound power (W), and extraction time (min) were considered independent variables, with optimization ranges of 0–500 W, 0–700 W, and 5–12 min, respectively. These variables were optimized using the response surface methodology (RSM) with a central composite design (CCD) [31,64]. The experimental designs for oregano, rosemary, hypericum, and chamomile are detailed in Table 7, Table 8, Table 9 and Table 10, respectively.

### 3.3. Characterization of Extracts

#### 3.3.1. The Extraction Yield (*Y*, %)

The extraction yield (*Y*, %) was expressed as the percentage of the weight of the dry extracts (*W_dry extract_*) obtained relative to the weight of the dry sample (*W_dry sample_*) used for extraction through Equation (25):

(25) $$Y\left(\%\right)=\frac{W_{dry\;extract}\;}{W_{dry\;sample}\;}\cdot 100,$$

#### 3.3.2. Antioxidant Activity (**IC_50_**)

The antioxidant activity of the extracts was quantified by assessing their ability to scavenge the free radical 2,2-diphenyl-1-picrylhydrazyl (DPPH), using the IC_50_ parameter. The IC_50_ value represents the concentration of extract solids required to scavenge 50% of the DPPH radical, and is expressed as the ratio of the mass of extract solids or pure substance to the initial mass of DPPH, in g/g DPPH.

To ensure accurate measurement, a preliminary dilution step was performed to achieve a final absorbance reading where A515 > 0.100. The extract was diluted to prepare a stock solution in a 25 mL volumetric flask, from which further dilutions (0.7C, 0.4C, and 0.1C) were prepared in duplicate.

The assay procedure involved adding 3.9 mL of DPPH to 0.1 mL of each dilution. The mixtures were vortexed and allowed to react for 30 min. Absorbance measurements were then taken at 515 nm using a Bel Photonics M51 UV–Vis Spectrometer (Bel Engineering s.r.l., Monza, Italy) with pure methanol used to zero the instrument [20,134]. The percentage of remaining DPPH (%rem DPPH) was calculated using Equation (26):

(26) $$DPPHrem\;\left(\%\right)=\frac{A_{t}\;}{A_{t=0}}\cdot 100,$$

where *A_t_* is the absorbance at 515 nm after 30 min of reaction, and *A_t_*_=0_ is the initial absorbance of the DPPH solution. The IC_50_ value, or the inhibition concentration at which 50% of the DPPH radical is scavenged, was determined by plotting the percentage of DPPH remaining against the concentration of the extract and interpolating the concentration required for 50% inhibition.

#### 3.3.3. Total Phenolic Content

The total phenolic content (TPC) of the extracts was determined using the Folin–Ciocalteu method, which expresses total phenolics as gallic acid equivalents (GAE). For each measurement, 7.9 mL of deionized water, 0.1 mL of the sample (or deionized water for the blank), 0.5 mL of Folin–Ciocalteu reagent, and 1.5 mL of saturated Na_2_CO_3_ solution were added sequentially to each test tube. After the addition of the Folin–Ciocalteu reagent, the tubes were incubated for 30 s to 8 min, vortexed, and then left to react in a shaded place for 2 h. Absorbance was measured at 765 nm using a Bel Photonics M51 UV–Vis Spectrometer (Bel Engineering s.r.l., Monza, Italy). To quantify the total phenolic content, a calibration curve was constructed using gallic acid as standard, with the x-axis representing the concentration of gallic acid and the y-axis representing the absorbance at 765 nm [135]. The TPC of the extracts was calculated based on the calibration curve and expressed in terms of gallic acid equivalents (mg GAE/g raw material).

#### 3.3.4. HPLC Analysis

The high-performance liquid chromatography with a diode array detector (HPLC–DAD) by Psarrou et al. (2020) [136], with some modifications, was used in order to detect the main compounds of the extracts. The HPLC apparatus consisted of a Prominence-i LC-2030 3D gradient pump and a diode array detector (Shimadzu, Kyoto, Japan). A Kromasil C18 column (5 µm, 250 × 4.6 mm, Nouryon AB, Göteborg, Sweden) was used under thermostated conditions at 30 °C. The samples were injected after filtration (0.45 μm, PVDF syringe filters, Teknokroma, Barcelona, Spain) and the flow rate was 1 mL/min. The solvent system consisted of water (A), methanol (B), acetonitrile (C) and isopropanol (D), each containing 0.2% trifluoroacetic acid. The initial composition of the mobile phase was 90% A, 6% B, 4% C and 0% D. With linear gradients, the composition changed to 85% A, 9% B, 6% C and 0% D within 5 min, 71% A, 17.4% B, 11.6% C and 0% D within 30 min, 0% A, 85% B, 15% C and 0% D within 60 min and 0% A, 60% B, 0% C and 40% D within 70 min. The injection volume was 20 µL, while the elution of compounds was monitored at 272, 280, 360, and 520 nm. System control, data acquisition, and data processing were performed using the LabSolutions Workstation (Shimadzu, Kyoto, Japan). The identification of compounds was performed using authentic reference analytical standards, matching retention times and UV–Vis spectra of the peaks, and comparing with literature data. The compounds were quantified using calibration curves generated from their respective authentic reference standards, including rosmarinic acid, carnosol, carnosic acid, carvacrol, rutin, quercetin, hyperforin, and hypericin. All calibration curves were obtained in the range of 20–200 mg/L analyzing four concentrations (20, 50, 100, 200 mg/L) from duplicate samples. The detection of compounds was performed at the previously mentioned wavelengths and the produced linear equations presented *R*^2^ > 0.998.

### 3.4. Experimental Design and Statistical Analysis

The response surface methodology (RSM) was used to analyze the experimental data, enabling the optimization of the extraction processes for each herb examined. This analysis utilized StatSoft STATISTICA 12.0 software (Hamburg, Germany). A Central Composite Design (CCD) was applied within the RSM framework to determine the optimal settings for the independent variables [60,62]. Each experiment was conducted in triplicate. The independent variables assessed included microwave power (W, *X*_1_), ultrasound power (W, *X*_2_), and extraction time (min, *X*_3_), with each variable coded at three levels: −1, 0, and 1. The dependent variables in the model consisted of the extraction yield (Y, %), IC_50_ (mg extract/g dried herb), and TPC (mg GAE/g dried herb). Table 1, Table 2, Table 3 and Table 4 display the responses obtained from 16 runs of the full experimental design for oregano, rosemary, hypericum, and chamomile. The generalized second-order quadratic equation that relates the response to the independent variables is as follows:

(27) $$Y=b_{0}+b_{1}X_{1}+b_{2}X_{2}+b_{3}X_{3}+b_{11}X_{1}^{2}+b_{22}X_{2}^{2}+b_{33}X_{3}^{2}+b_{12}X_{1}X_{2}+b_{13}X_{1}X_{3}+b_{23}X_{2}X_{3}$$

where *Y* is the dependent variable (extraction yield, IC_50_, TPC), *b*_0_ is the intercept, *b*_1_, *b*_2_, and *b*_3_ are the linear coefficients, *b*_11_, *b*_22_, and *b*_33_ are the quadratic coefficients, and *b*_12_, *b*_13_, *b*_23_ represent the interaction effects between variables [62,64]. The independent variables *X*_1_, *X*_2_, and *X*_3_ correspond to microwave power, ultrasound power and extraction time, respectively.

An analysis of variance (ANOVA) was used to assess the significance (*p* < 0.05) of the independent variables for the extraction yield, IC_50_, and TPC. The variable with the lowest *p*-value was considered to have the most significant impact (*p* < 0.05) on the response. Non-significant variables (*p* > 0.05) were excluded from the model, unless the quadratic or interaction effects were significant alongside that variable. The reduced model was then used to interpret the data, removing the non-significant factors.

A significance level of 95% was used for the error criteria. ANOVA also determined the significance level for each response, while a lack-of-fit test assessed the suitability of the mathematical model, using the F-value to indicate how well the model fit the data. The model’s predictive accuracy was evaluated through the *R*^2^ value. Three-dimensional surface plots were used to visualize the effects of independent variables and their interactions [64]. For clarity purposes, the interaction effects of factors with the greatest impact on dependent variables (extraction yield, IC_50_, TPC) are presented.

Moreover, one-way ANOVA was employed to compare differences between the samples. Tukey’s post-hoc test (α = 0.05) was applied to identify significant differences, and all statistical analyses were conducted using the StatSoft STATISTICA 12.0 software (Hamburg, Germany).

## 4. Conclusions

This study demonstrated the successful optimization of the synergistic combination of UAE–MAE in efficiently recovering bioactive compounds from oregano (*Origanum vulgare*), rosemary (*Rosmarinus officinalis*), hypericum (*Hypericum perforatum*), and chamomile (*Matricaria recutita*). By employing ethanol as a green solvent and optimizing key extraction parameters using the response surface methodology (RSM), the research achieved significant improvements in extraction yields, antioxidant activity, and total phenolic content (TPC). This study highlights the variability in optimal conditions for each plant, underscoring the importance of tailored approaches. Oregano exhibited strong antioxidant activity (IC_50_ 50.31 mg extract/g) and the highest TPC (34.99 mg GAE/g) under 500 W MW, 700 W US, and 12 min, while rosemary achieved the highest extraction yield (23.36%) under 200 W MW, 700 W US, and 8 min. Hypericum demonstrated the strongest antioxidant activity (IC_50_ 29.8 mg extract/g) and TPC (53.7 mg GAE/g) under moderate US power and extraction times (200 W MW, 450 W US, 12 min). Chamomile, though achieving a respectable yield (17.79%), showed relatively weaker antioxidant properties and TPC, reflecting its distinct bioactive profile. These findings illustrate that combining UAE and MAE leverages the complementary strengths of both techniques, yielding phenolic-rich extracts while minimizing compound degradation. The simultaneous integration of UAE’s mechanical disruption with MAE’s rapid heating into a single, optimized protocol overcomes the limitations of conventional and single-mode extractions. The systematic optimization ensured precise control over parameters, enhancing compound recovery, preserving bioactive integrity, and minimizing energy and solvent consumption. By achieving yields and bioactive recovery rates that match or exceed literature benchmarks, this study highlights the potential of UAE–MAE for industrial applications. The sustainable use of locally abundant medicinal plants supports regional bioeconomies and environmental conservation efforts. The findings have broad implications for the food, nutraceutical, and pharmaceutical industries, where high-quality, phenolic-rich extracts are increasingly in demand. This work sets a foundation for future research into UAE–MAE combinations across diverse plant matrices, promoting innovative, green technologies for the efficient utilization of natural resources.

## Figures and Tables

**Table 1:** Analysis of variance for the response surface of Y (%), IC_50_ (mg extract/g raw material), and TPC (mg GAE/g raw material) for oregano extracts.

Source	Coefficients	Standard Error	Sum of Squares	DF	Mean Square	F-Value
*Y* (%) ^a^						
Model	7.85	2.30	4306.56	10	430.66	357.63 ***
*X*_1_	1.51 × 10^−^^2^	4.20 × 10^−^^3^	154.57	1	154.57	128.36 ***
*X*_1_^2^	−2.54 × 10^−^^5^	7.00 × 10^−^^6^	17.79	1	17.79	14.78 ***
*X*_2_	1.37 × 10^−^^3^	2.90 × 10^−^^3^	22.76	1	22.76	18.90 ***
*X*_2_^2^	−1.79 × 10^−^^6^	4.00 × 10^−^^6^	0.31	1	0.31	0.25 ^NS^
*X*_3_	−1.28	5.80 × 10^−^^1^	274.63	1	274.63	228.07 ***
*X*_3_^2^	8.76 × 10^−^^2^	3.30 × 10^−^^2^	8.48	1	8.48	7.04 **
*X*_1_ *X*_2_	−1.75 × 10^−^^5^	3.00 × 10^−^^6^	57.57	1	57.57	47.81 ***
*X*_1_ *X*_3_	1.51 × 10^−^^3^	2.50 × 10^−^^4^	42.44	1	42.44	35.25 ***
*X*_2_ *X*_3_	7.94 × 10^−^^4^	1.80 × 10^−^^4^	23.18	1	23.18	19.25 ***
Residual			42.15	35	1.20	
Total			628.42	44		
**IC_50_ (mg extract/g raw material) ^b^**						
Model	108.41	5.99	157,051.70	10	15,705.17	1926.29 ***
*X*_1_	1.34 × 10^−^^1^	1.09 × 10^−^^2^	280.30	1	280.30	34.38 ***
*X*_1_^2^	−3.00 × 10^−^^4^	1.70 × 10^−^^5^	3365.08	1	3365.08	412.74 ***
*X*_2_	1.04 × 10^−^^1^	7.70 × 10^−^^3^	278.17	1	278.17	34.12 ***
*X*_2_^2^	−1.00 × 10^−^^4^	9.00 × 10^−^^6^	1949.11	1	1949.11	239.06 ***
*X*_3_	−1.54 × 10^1^	1.50	19.08	1	19.08	2.34 ^NS^
*X*_3_^2^	8.51 × 10^−^^1^	8.60 × 10^−^^2^	799.77	1	799.77	98.09 ***
*X*_1_ *X*_2_	0.00	7.00 × 10^−^^6^	35.69	1	35.69	4.38 *
*X*_1_ *X*_3_	2.90 × 10^−^^3^	6.60 × 10^−^^4^	152.09	1	152.09	18.65 ***
*X*_2_ *X*_3_	1.00 × 10^−^^3^	4.70 × 10^−^^4^	0.30	1	0.30	0.036 ^NS^
Residual			285.39	35	8.15	
Total			9396.47	44		
**TPC (mg GAE/g raw material) ^c^**						
Model	39.09	6.93	63,222.86	10	6322.29	579.48 ***
*X*_1_	1.57 × 10^−^^1^	1.26 × 10^−^^2^	297.781	1	297.78	27.29 ***
*X*_1_^2^	−1.90 × 10^−^^4^	2.00 × 10^−^^5^	1037.66	1	1037.66	95.11 ***
*X*_2_	4.89 × 10^−^^2^	8.90 × 10^−^^3^	1309.18	1	1309.18	120.00 ***
*X*_2_^2^	−4.00 × 10^−^^5^	1.10 × 10^−^^5^	160.16	1	160.16	14.68 ***
*X*_3_	−6.34 × 10^0^	1.73	20.73	1	20.73	1.90 ^NS^
*X*_3_^2^	3.99 × 10^−^^1^	9.90 × 10^−^^2^	176.63	1	176.63	16.19 ***
*X*_1_ *X*_2_	−4.00 × 10^−^^5^	8.00 × 10^−^^6^	260.06	1	260.06	23.84 ***
*X*_1_ *X*_3_	−4.07 × 10^−^^3^	7.70 × 10^−^^4^	308.66	1	308.66	28.29 ***
*X*_2_ *X*_3_	9.40 × 10^−^^4^	5.50 × 10^−^^4^	32.21	1	32.21	2.95 ^NS^
Residual			381.86	35	10.91	
Total				44		

* *p* < 0.05, ** *p* < 0.01, and *** *p* < 0.001. ^NS^—not significant. ^a^ The coefficient of determination (*R*^2^) of the model was 0.93. ^b^ The coefficient of determination (*R*^2^) of the model was 0.97. ^c^ The coefficient of determination (*R*^2^) of the model was 0.91.

**Table 2:** Analysis of variance for the response surface of *Y* (%), IC_50_ (mg extract/g raw material), and TPC (mg GAE/g raw material) for rosemary extracts.

Source	Coefficients	Standard Error	Sum of Squares	DF	Mean Square	F-Value
*Y* (%) ^a^						
Model	−16.00	2.97	10,542.36	10	1054.24	525.85 ***
*X*_1_	3.29 × 10^−^^2^	5.42 × 10^−^^3^	72.99	1	72.99	36.41 ***
*X*_1_^2^	−4.66 × 10^−^^5^	9.00 × 10^−^^6^	59.68	1	59.68	29.77 ***
*X*_2_	−3.00 × 10^−^^2^	3.80 × 10^−^³	282.58	1	282.58	140.95 ***
*X*_2_^2^	5.14 × 10^−^^5^	5.00 × 10^−^^6^	252.52	1	252.52	125.96 ***
*X*_3_	6.41 × 10^0^	7.42 × 10^−^^1^	141.30	1	141.30	70.48 ***
*X*_3_^2^	−3.39 × 10^−^^1^	4.26 × 10^−^^2^	126.89	1	126.89	63.29 ***
*X*_1_ *X*_2_	2.13 × 10^−^^6^	3.00 × 10^−^^6^	0.85	1	0.85	0.43 ^NS^
*X*_1_ *X*_3_	−4.81 × 10^−^^4^	3.28 × 10^−^^4^	4.30	1	4.30	2.15 ^NS^
*X*_2_ *X*_3_	2.69 × 10^−^^4^	2.34 × 10^−^^4^	2.66	1	2.66	1.33 ^NS^
Residual			70.17	35	2.00	
Total			853.41	44		
**IC_50_ (mg extract/g raw material) ^b^**						
Model	58.80	6.89	91,356.59	10	9135.66	845.44 ***
*X*_1_	−2.40 × 10^−^^2^	1.26 × 10^−^^2^	3974.57	1	3974.57	367.82 ***
*X*_1_^2^	1.95 × 10^−^^5^	2.00 × 10^−^^5^	10.40	1	10.40	0.96 ^NS^
*X*_2_	−5.31 × 10^−^³	8.82 × 10^−^³	2263.64	1	2263.64	209.48 ***
*X*_2_^2^	−3.61 × 10^−^^5^	1.10 × 10^−^^5^	124.39	1	124.39	11.51 **
*X*_3_	−6.10 × 10^0^	1.72 × 10^0^	193.00	1	193.00	17.86 ***
*X*_3_^2^	3.22 × 10^−^^1^	9.89 × 10^−^^2^	114.43	1	114.43	10.59 **
*X*_1_ *X*_2_	1.31 × 10^−^^4^	8.00 × 10^−^⁶	3243.53	1	3243.53	300.16 ***
*X*_1_ *X*_3_	1.70 × 10^−^³	7.62 × 10^−^^4^	53.68	1	53.68	4.97 *
*X*_2_ *X*_3_	2.66 × 10^−^³	5.42 × 10^−^^4^	259.63	1	259.63	24.03 ***
Residual			378.20	35	10.81	
Total			10,985.13	44		
**TPC (mg GAE/g raw material) ^c^**						
Model	13.50	4.03	24,638.06	10	2463.81	666.99 ***
*X*_1_	2.92 × 10^−^^2^	7.35 × 10^−^^3^	251.75	1	251.75	68.15 ***
*X*_1_^2^	−4.05 × 10^−^^5^	1.20 × 10^−^^5^	45.12	1	45.11	12.21 **
*X*_2_	−2.07 × 10^−^^2^	5.16 × 10^−^^3^	465.08	1	465.08	125.91 ***
*X*_2_^2^	3.33 × 10^−^^6^	6 × 10^−^⁶	1.06	1	1.06	0.29 ^NS^
*X*_3_	1.70	1.01	25.59	1	25.58	6.93 *
*X*_3_^2^	−1.37 × 10^−^^1^	5.78 × 10^−^^2^	20.70	1	20.70	5.60 *
*X*_1_ *X*_2_	1.95 × 10^−^^5^	4 × 10^−^^6^	71.64	1	71.64	19.39 ***
*X*_1_ *X*_3_	−4.92 × 10^−^^4^	4.46 × 10^−^^4^	4.50	1	4.50	1.22 ^NS^
*X*_2_ *X*_3_	2.91 × 10^−^³	3.17 × 10^−^^4^	310.98	1	310.98	84.19 ***
Residual			129.29	35	3.69	
Total				44		

* *p* < 0.05, ** *p* < 0.01, and *** *p* < 0.001. ^NS^—not significant. ^a^ The coefficient of determination (*R*^2^) of the model was 0.92. ^b^ The coefficient of determination (*R*^2^) of the model was 0.97. ^c^ The coefficient of determination (*R*^2^) of the model was 0.91.

**Table 3:** Analysis of variance for the response surface of *Y* (%), IC_50_ (mg extract/g raw material), and TPC (mg GAE/g raw material) for hypericum extracts.

Source	Coefficients	Standard Error	Sum of Squares	DF	Mean Square	F-Value
*Y* (%) ^a^						
Model	6.60	2.27	7419.22	10	741.92	630.91 ***
*X*_1_	4.31	4.10 × 10^−3^	475.56	1	475.56	404.40 ***
*X*_1_^2^	−4.65	7.00 × 10^−6^	59.49	1	59.49	50.59 ***
*X*_2_	4.98	2.90 × 10^−3^	38.21	1	38.21	32.49 ***
*X*_2_^2^	5.39	4.00 × 10^−6^	2.78	1	2.78	2.36 ^NS^
*X*_3_	−6.16	7.00 × 10^−6^	13.83	1	13.83	11.76 *
*X*_3_^2^	4.08	3.30 × 10^−2^	1.84	1	1.84	1.56 ^NS^
*X*_1_ *X*_2_	−2.22	2.00 × 10^−5^	93.14	1	93.14	79.20 ***
*X*_1_ *X*_3_	4.62	2.50 × 10^−4^	3.96	1	3.96	3.37 ^NS^
*X*_2_ *X*_3_	4.11	1.80 × 10^−6^	6.00 × 10^−4^	1	6 × 10^−4^	5 × 10^−4 NS^
Residual			41.16	35	1.18	
Total			678.59	44		
**IC_50_ (mg extract/g raw material) ^b^**						
Model	5.84	24.27	248,786.70	10	24,878.67	185.71 ***
*X*_1_	1.56 × 10^−2^	4.40 × 10^−2^	2811.89	1	2811.89	20.99 ***
*X*_1_^2^	3.39 × 10^−4^	7.00 × 10^−5^	3153.69	1	3153.69	23.54 ***
*X*_2_	−1.47 × 10^−1^	3.10 × 10^−2^	11,365.17	1	11,365.17	84.84 ***
*X*_2_^2^	2.54 × 10^−4^	4.00 × 10^−4^	6170.95	1	6170.95	46.064 ***
*X*_3_	−1.78	6.10 × 10^−1^	1156.35	1	1156.35	8.63 **
*X*_3_^2^	4.45 × 10^−1^	3.50 × 10^−1^	218.88	1	218.88	1.63 ^NS^
*X*_1_ *X*_2_	−1.88 × 10^−4^	3.00 × 10^−4^	6617.12	1	6617.12	49.39 ***
*X*_1_ *X*_3_	−9.48 × 10^−3^	7.00 × 10^−5^	1670.89	1	1670.89	12.47 **
*X*_2_ *X*_3_	−4.67 × 10^−3^	1.90 × 10^−3^	802.02	1	802.02	5.99 *
Residual			4688.81	35	133.97	
Total			53,028.48	44		
**TPC (mg GAE/g raw material) ^c^**						
Model	−9.94	5.70	79,125.48	10	7912.55	1051.17 ***
*X*_1_	5.15 × 10^−2^	1.00 × 10^−2^	642.46	1	642.46	85.35 ***
*X*_1_^2^	−6.31 × 10^−5^	1.70 × 10^−5^	109.57	1	109.57	14.56 ***
*X*_2_	7.39 × 10^−2^	7.40 × 10^−3^	1089.64	1	1089.64	144.76 ***
*X*_2_^2^	−8.32 × 10^−5^	9.00 × 10^−5^	661.90	1	661.90	87.93 ***
*X*_3_	8.26 × 10^0^	1.40 × 10^−1^	322.66	1	322.66	42.86 ***
*X*_3_^2^	−4.16 × 10^−1^	8.30 × 10^−2^	191.28	1	191.28	25.41 ***
*X*_1_ *X*_2_	1.30 × 10^−5^	6.00 × 10^−6^	31.84	1	31.84	4.23 *
*X*_1_ *X*_3_	−7.00 × 10^−4^	6.40 × 10^−4^	9.12	1	9.12	1.21 ^NS^
*X*_2_ *X*_3_	−1.95 × 10^−4^	4.50 × 10^−4^	1.39	1	1.40	0.18 ^NS^
Residual			263.46	35	7.53	
Total			4643.40	44		

* *p* < 0.05, ** *p* < 0.01, and *** *p* < 0.001. ^NS^—not significant. ^a^ The coefficient of determination (*R*^2^) of the model was 0.94. ^b^ The coefficient of determination (*R*^2^) of the model was 0.92. ^c^ The coefficient of determination (*R*^2^) of the model was 0.94.

**Table 4:** Analysis of variance for the response surface of *Y* (%), IC_50_ (mg extract/g raw material), and TPC (mg GAE/g raw material) for chamomile extracts.

Source	Coefficients	Standard Error	Sum of Squares	DF	Mean Square	F-Value
*Y* (%) ^a^						
Model	148.00	2.69 × 10^0^	10,858.42	10	1085.84	661.14 ***
*X*_1_	2.85 × 10^−^^2^	4.90 × 10^−^^3^	251.69	1	251.69	153.25 ***
*X*_1_^2^	−2.17 × 10^−^^5^	8.00 × 10^−^^6^	12.92	1	12.92	7.8675 **
*X*_2_	1.14 × 10^−^^2^	3.40 × 10^−^^3^	322.79	1	322.79	196.54 ***
*X*_2_^2^	3.01 × 10^−^^6^	4.00 × 10^−^^6^	0.86	1	0.86	0.53 ^NS^
*X*_3_	−3.04 × 10^0^	6.72 × 10^−^^1^	211.42	1	211.43	128.73 ***
*X*_3_^2^	2.14 × 10^−^^1^	3.90 × 10^−^^2^	50.77	1	50.77	30.91 ***
*X*_1_ *X*_2_	−2.46 × 10^−^^5^	3.00 × 10^−^^6^	113.69	1	113.69	69.22 ***
*X*_1_ *X*_3_	3.03 × 10^−^^4^	2.97 × 10^−^^4^	1.71	1	1.71	1.04 ^NS^
*X*_2_ *X*_3_	2.41 × 10^−^^4^	2.11 × 10^−^^4^	2.13	1	2.13	1.30 ^NS^
Residual			57.48	35	1.64	
Total			1029.07	44		
**IC_50_ (mg extract/g raw material) ^b^**						
Model	241.00	16.00	558,122.00	10	55,812.20	961.60 ***
*X*_1_	−2.39 × 10^−^^1^	2.91 × 10^−^^2^	198.00	1	197.96	3.41 ^NS^
*X*_1_^2^	6.14 × 10^−^^5^	5.00 × 10^−^^5^	103.50	1	103.53	1.78 ^NS^
*X*_2_	−3.67 × 10^−^^1^	2.04 × 10^−^^2^	77,410.50	1	77,410.50	1333.72 ***
*X*_2_^2^	2.50 × 10^−^^4^	2.00 × 10^−^^5^	5968.00	1	5967.99	102.82 ***
*X*_3_	−1.65 × 10^1^	3.99 × 10^0^	8219.40	1	8219.39	141.61 ***
*X*_3_^2^	1.08 × 10^0^	2.29 × 10^−^^1^	1280.60	1	1280.64	22.06 ***
*X*_1_ *X*_2_	2.35 × 10^−^^4^	2.00 × 10^−^^5^	10,355.50	1	10,355.52	178.42 ***
*X*_1_ *X*_3_	1.36 × 10^−^^2^	1.77 × 10^−^^3^	3463.90	1	3463.85	59.68 ***
*X*_2_ *X*_3_	−1.38 × 10^−^^3^	1.26 × 10^−^^3^	70.00	1	70.00	1.21 ^NS^
Residual			2031.40	35	58.04	
Total			131,476.80	44		
**TPC (mg GAE/g raw material) ^c^**						
Model	4.02	3.30	4503.69	10	450.37	181.89 ***
*X*_1_	6.18 × 10^−^^3^	6.02 × 10^−^^3^	348.64	1	348.64	140.81 ***
*X*_1_^2^	2.24 × 10^−^^5^	9.00 × 10^−^^6^	13.84	1	13.84	5.59 *
*X*_2_	2.78 × 10^−^^2^	4.22 × 10^−^^3^	356.63	1	356.63	144.03 ***
*X*_2_^2^	−2.03 × 10^−^^5^	5.00 × 10^−^^6^	39.24	1	39.23	15.85 ***
*X*_3_	−8.13 × 10^−^^1^	8.25 × 10^−^^1^	1.81	1	1.81	0.73 ^NS^
*X*_3_^2^	3.60 × 10^−^^2^	4.73 × 10^−^^2^	1.43	1	1.43	0.58 ^NS^
*X*_1_ *X*_2_	−1.91 × 10^−^^5^	4.00 × 10^6^	68.45	1	68.45	27.65 ***
*X*_1_ *X*_3_	3.46 × 10^−^^4^	3.65 × 10^−^^4^	2.23	1	2.23	0.90 ^NS^
*X*_2_ *X*_3_	1.25 × 10^−^^4^	2.60 × 10^−^^4^	0.58	1	0.58	0.23 ^NS^
Residual			86.66	35	2.48	
Total			953.47	44		

* *p* < 0.05, ** *p* < 0.01, and *** *p* < 0.001. ^NS^—not significant. ^a^ The coefficient of determination (*R*^2^) of the model was 0.94. ^b^ The coefficient of determination (*R*^2^) of the model was 0.98. ^c^ The coefficient of determination (*R*^2^) of the model was 0.91.

**Table 5:** Optimized extraction conditions and corresponding responses of the investigated plants.

Plant	UAE (W)	MAE (W)	Time (min)	Y (%)	TPC (mg GAE/g Dry Sample)	IC_50_ (mg Extract/g Dry Sample)
Oregano *(Origanum vulgare)*	700	500	12	16.57 ± 1.06 ^b^	34.99 ± 1.04 ^b^	50.31 ± 1.89 ^b^
Rosemary (*Rosmarinus officinalis*)	700	200	8	23.36 ± 0.17 ^a^	26.35 ± 1.03 ^c^	40.75 ± 1.11 ^c^
Hypericum (*Hypericum perforatum*)	450	200	12	14.49 ± 0.40 ^c^	53.67 ± 1.32 ^a^	29.78 ± 1.25 ^d^
Chamomile (*Matricaria recutita*)	700	500	5	17.79 ± 0.12 ^b^	12.85 ± 1.23 ^d^	55.82 ± 2.84 ^a^

Different superscript letters indicate significant differences among the TPC, IC_50_, and Y (%) values for the investigated plants under the optimized extraction conditions for each plant (*p* < 0.05).

**Table 6:** Quantification of key bioactive compounds in optimized extracts.

Plant	Compound	Concentration (mg/g Dry Sample)
Oregano *(Origanum vulgare)*	Carvacrol	10.1 ± 0.4
	Rosmarinic acid	9.2 ± 0.6
Rosemary *(Rosmarinus officinalis)*	Carnosic acid	6.6 ± 0.2
	Carnosol	2.3 ± 0.2
	Rosmarinic acid	5.0 ± 0.3
Hypericum (*Hypericum perforatum*)	Hyperforin	10.4 ± 0.5
	Hypericin	2.1 ± 0.2
Chamomile (*Matricaria recutita*)	Querquetin	3.5 ± 0.4
	Rutin	2.5 ± 0.3

**Table 7:** Extraction yield (%), IC_50_ (mg extract/g raw material), and TPC (mg GAE/g raw material) (dependent variables) responses of oregano extracts based on central composite design.

	Variable Levels			Observed Values		
Run	*X*_1_ (Microwave Power, W)	*X*_2_ (Ultrasound Power, W)	*X*_3_ (Extraction Time, Min)	Y (%)	IC_50_ (mg Extract/g Dried Herb)	TPC (mg GAE/g Dried Herb)
1	0	0	5	3.26 ^g^ ± 0.59	52.36 ^d,e,f^ ± 2.16	17.07 ^i^ ± 1.21
2	0	0	12	4.43 ^f,g^ ± 0.79	45.91 ^f,g^ ± 3.58	18.77 ^i^ ± 0.57
3	0	450	8	7.75 ^d,e^ ± 0.62	55.74 ^c,d,e^ ± 2.07	30.17 ^g,h^ ± 1.05
4	0	700	5	6.43 ^e,f^ ± 0.15	57.06 ^c,d,e^ ± 2.35	37.77 ^d,e,f^ ± 1.06
5	0	700	12	11.15 ^c^ ± 0.62	50.39 ^e,f^ ± 2.41	42.50 ^b,c,d^ ± 1.39
6	200	0	8	7.66 ^d,e^ ± 0.88	58.13 ^c,d^ ± 1.72	34.61 ^f,g^ ± 1.42
7	200	450	5	6.77 ^e,f^ ± 0.45	88.67 ^a^ ± 2.63	41.78 ^b,c^ ± 3.85
8	200	450	8	10.15 ^c^ ± 0.46	73.65 ^b^ ± 2.57	47.35 ^a,b^ ± 2.41
9	200	450	8	10.15 ^c^ ± 0.46	73.65 ^b^ ± 2.57	47.35 ^a,b^ ± 2.41
10	200	450	12	14.10 ^b^ ± 1.04	85.32 ^a^ ± 2.87	53.00 ^a^ ± 2.73
11	200	700	8	9.23 ^c,d^ ± 0.28	62.07 ^c^ ± 3.21	40.59 ^c,d,e^ ± 2.76
12	500	0	5	9.20 ^c,d^ ± 0.57	38.11 ^h^ ± 2.01	36.92 ^d,e,f^ ± 1.15
13	500	0	12	15.39 ^a,b^ ± 0.96	41.36 ^g,h^ ± 3.19	25.19 ^h^ ± 1.37
14	500	450	8	7.75 ^d,e^ ± 0.51	54.97 ^c,d,e^ ± 2.54	35.40 ^e,f,g^ ± 3.02
15	500	700	5	6.08 ^e,f^ ± 0.12	46.03 ^f,g^ ± 2.02	43.84 ^b,c^ ± 1.39
16	500	700	12	16.57 ^a^ ± 1.06	50.31 ^e,f^ ± 1.89	34.99 ^e,f,g^ ± 1.04

Values labeled with different letters indicate significant differences (*p* < 0.05).

**Table 8:** Extraction yield (%), IC_50_ (mg extract/g raw material), and TPC (mg GAE/g raw material) (dependent variables) responses of rosemary extracts based on central composite design.

	Variable Levels			Observed Values		
Run	*X*_1_ (Microwave Power, W)	*X*_2_ (Ultrasound Power, W)	*X*_3_ (Extraction Time, Min)	Y (%)	IC_50_ (mg Extract/g Dried Herb)	TPC (mg GAE/g Dried Herb)
1	0	0	5	7.28 ^l^ ± 0.07	35.14 ^e^ ± 1.64	16.38 ^f,g,h^ ± 0.86
2	0	0	12	10.88 ^j^ ± 0.08	34.68 ^e^ ± 0.98	15.52 ^g,h^ ± 0.42
3	0	450	8	13.92 ^g^ ± 0.08	25.51 ^h^ ± 0.53	19.50 ^e,f^ ± 1.09
4	0	700	5	11.79 ^i^ ± 0.19	28.67 ^f,g^ ± 1.02	17.86 ^f,g^ ± 1.18
5	0	700	12	18.48 ^d^ ± 0.09	31.90 ^e,f^ ± 0.93	25.23 ^b,c,d^ ± 0.81
6	200	0	8	19.09 ^c^ ± 0.09	29.69 ^f,g^ ± 1.09	23.53 ^c,d^ ± 0.91
7	200	450	5	9.94 ^k^ ± 0.15	41.48 ^d^ ± 1.01	23.05 ^c,d^ ± 1.02
8	200	450	8	12.74 ^h^ ± 0.14	41.07 ^d^ ± 2.11	24.95 ^b,c,d^ ± 1.38
9	200	450	8	12.74 ^h^ ± 0.14	41.07 ^d^ ± 2.11	24.95 ^b,c,d^ ± 1.38
10	200	450	12	15.19 ^e^ ± 0.12	49.10 ^c^ ± 2.28	25.13 ^b,c,d^ ± 1.32
11	200	700	8	23.36 ^a^ ± 0.17	40.75 ^d^ ± 1.11	26.35 ^b,c^ ± 1.03
12	500	0	5	10.70 ^j^ ± 0.10	34.68 ^e^ ± 1.12	22.62 ^d,e^ ± 1.24
13	500	0	12	14.44 ^f^ ± 0.10	31.90 ^e,f^ ± 0.65	13.88 ^h^ ± 0.71
14	500	450	8	13.76 ^g^ ± 0.14	64.61 ^b^ ± 2.83	27.91 ^b^ ± 1.31
15	500	700	5	18.24 ^d^ ± 0.11	63.56 ^b^ ± 1.05	24.30 ^c,d^ ± 1.21
16	500	700	12	21.08 ^b^ ± 0.10	82.67 ^a^ ± 1.07	35.92 ^a^ ± 2.02

Values labeled with different letters indicate significant differences (*p* < 0.05).

**Table 9:** Extraction yield (%), IC_50_ (mg extract/g raw material), and TPC (mg GAE/g raw material) (dependent variables) responses of hypericum extracts based on central composite design.

	Variable Levels			Observed Values		
Run	*X*_1_ (Microwave Power, W)	*X*_2_ (Ultrasound Power, W)	*X*_3_ (Extraction Time, Min)	Y (%)	IC_50_ (mg Extract/g Dried Herb)	TPC (mg GAE/g Dried Herb)
1	0	0	5	3.98 ^j^ ± 0.29	74.94 ^d^ ± 2.62	18.91 ^i^ ± 0.53
2	0	0	12	5.50 ^i^ ± 0.30	96.81 ^c^ ± 3.68	28.35 ^h^ ± 1.14
3	0	450	8	9.43 ^h^ ± 0.38	30.33 ^h^ ± 1.17	45.92 ^c,d^ ± 1.49
4	0	700	5	10.35 ^f,g^ ± 0.21	53.12 ^f^ ± 2.41	33.54 ^g^ ± 0.91
5	0	700	12	9.92 ^g,h^ ± 0.30	96.81 ^c^ ± 3.68	38.35 ^f^ ± 1.14
6	200	0	8	10.91 ^f^ ± 0.23	62.87 ^e^ ± 2.45	41.03 ^e,f^ ± 2.43
7	200	450	5	12.80 ^e^ ± 0.27	31.98 ^h^ ± 1.02	44.76 ^d,e^ ± 2.16
8	200	450	8	13.33 ^d,e^ ± 0.27	30.02 ^h^ ± 1.19	50.13 ^b,c^ ± 2.38
9	200	450	8	13.33 ^d,e^ ± 0.27	30.02 ^h^ ± 1.19	50.13 ^b,c^ ± 2.38
10	200	450	12	14.49 ^c^ ± 0.40	29.78 ^h^ ± 1.25	53.67 ^a,b^ ± 1.32
11	200	700	8	15.58 ^b^ ± 0.36	52.39 ^f^ ± 1.98	44.37 ^d,e^ ± 1.26
12	500	0	5	16.75 ^a^ ± 0.28	118.75 ^b^ ± 2.97	28.83 ^g,h^ ± 0.83
13	500	0	12	17.64 ^a^ ± 0.32	148.44 ^a^ ± 3.71	33.33 ^g^ ± 1.21
14	500	450	8	12.48 ^e^ ± 0.30	62.10 ^e^ ± 2.13	55.98 ^a^ ± 1.72
15	500	700	5	14.00 ^c,d^ ± 0.34	71.75 ^d^ ± 2.01	44.41 ^d,e^ ± 2.22
16	500	700	12	16.99 ^a^ ± 0.26	41.02 ^g^ ± 1.09	49.75 ^b,c^ ± 1.34

Values labeled with different letters indicate significant differences (*p* < 0.05).

**Table 10:** Extraction yield (%), IC_50_ (mg extract/g raw material), and TPC (mg GAE/g raw material) (dependent variables) responses of chamomile extracts based on central composite design.

	Variable Levels			Observed Values		
Run	*X*_1_ (Microwave Power, W)	*X*_2_ (Ultrasound Power, W)	*X*_3_ (Extraction Time, Min)	Y (%)	IC_50_ (mg Extract/g Dried herb)	TPC (mg GAE/g Dried Herb)
1	0	0	5	6.38 ^l^ ± 0.08	192.00 ^a^ ± 5.14	0.45 ^f^ ± 0.10
2	0	0	12	7.49 ^k^ ± 0.05	200.14 ^a^ ± 5.42	0.60 ^f^ ± 0.10
3	0	450	8	10.58 ^i^ ± 0.15	48.34 ^i^ ± 1.07	9.56 ^d,e^ ± 1.19
4	0	700	5	14.86 ^f^ ± 0.16	51.42 ^h,i^ ± 2.18	10.98 ^b,c,d,e^ ± 1.20
5	0	700	12	21.17 ^a^ ± 0.12	49.57 ^i^ ± 1.08	8.16 ^e^ ± 1.02
6	200	0	8	9.78 ^j^ ± 0.09	146.87 ^c^ ± 3.47	0.68 ^f^ ± 0.11
7	200	450	5	12.34 ^h^ ± 0.13	47.12 ^i^ ± 1.04	9.70 ^c,d,e^ ± 1.08
8	200	450	8	13.70 ^g^ ± 0.06	60.58 ^g,h^ ± 2.65	9.91 ^c,d,e^ ± 1.08
9	200	450	8	13.70 ^g^ ± 0.06	60.58 ^g,h^ ± 2.65	9.91 ^c,d,e^ ± 1.08
10	200	450	12	21.30 ^a^ ± 0.16	93.00 ^f^ ± 3.04	10.20 ^c,d,e^ ± 1.20
11	200	700	8	16.52 ^d^ ± 0.14	50.97 ^i^ ± 1.10	11.72 ^b,c,d^ ± 1.21
12	500	0	5	13.44 ^g^ ± 0.11	117.14 ^d^ ± 4.06	12.05 ^a,b,c,d^ ± 1.14
13	500	0	12	20.58 ^b^ ± 0.12	174.57 ^b^ ± 4.21	9.68 ^d,e^ ± 1.13
14	500	450	8	15.44 ^e^ ± 0.09	70.01 ^g^ ± 2.11	13.40 ^a,b^ ± 1.24
15	500	700	5	17.79 ^c^ ± 0.12	55.82 ^h,i^ ± 2.84	12.85 ^a,b,c^ ± 1.23
16	500	700	12	20.96 ^a^ ± 0.16	106.49 ^e^ ± 3.52	14.89 ^a^ ± 1.32

Values labeled with different letters indicate significant differences (*p* < 0.05).

## Data Availability

Data are contained within this article and Supplementary Materials.

## Supplementary Materials

The following supporting information can be downloaded at: https://www.mdpi.com/article/10.3390/molecules29235773/s1, Table S1: Spectra of compounds identified with HPLC analysis of oregano extract; Table S2: UV Spectra of compounds identified with HPLC analysis of rosemary extract; Table S3: UV Spectra of compounds identified with HPLC analysis of hypericum extract, Table S4: UV Spectra of compounds identified with HPLC analysis of chamomile extract.
