# Supplementary material for: Optimization of Combined Ultrasound and Microwave-Assisted Extraction for Enhanced Bioactive Compounds Recovery from Four Medicinal Plants: Oregano, Rosemary, Hypericum, and Chamomile
Source: Molecules. 2024 Dec 6;29(23):5773. doi: 10.3390/molecules29235773 (PMC11643802; doi:10.3390/molecules29235773)
Supplement: Supplementary file 1 [file molecules-29-05773-s001.zip › molecules-3304892-supplementary.pdf]

Table S1 Spectra of compounds identified with HPLC analysis of oregano extract

Compound	UV Spectrum
Carvacrol	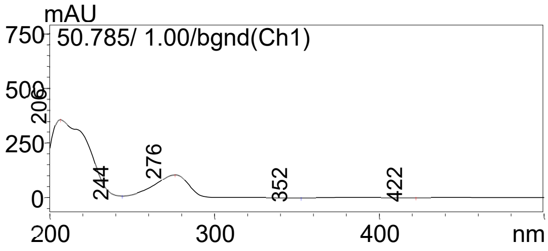
Flavonoid 1	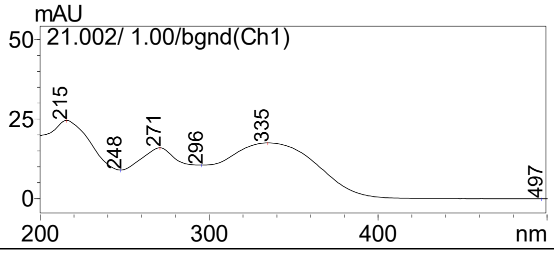
Flavonoid 2	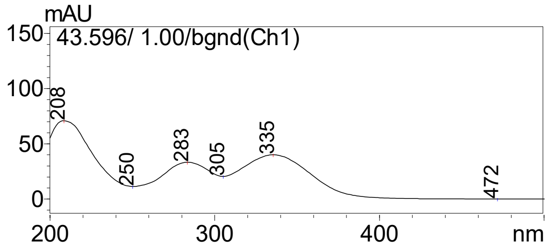
Flavonoid 3	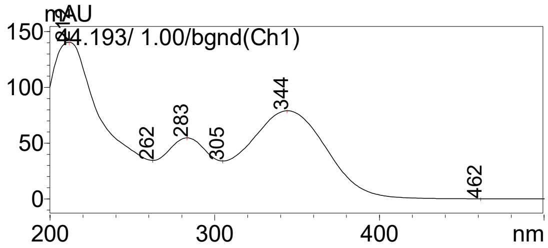
Flavonoid 4	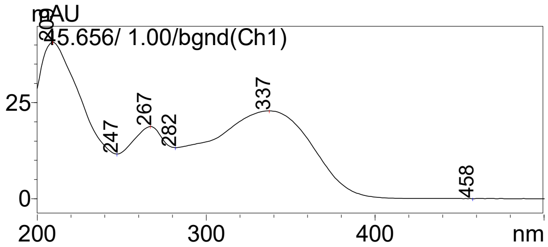
Flavonoid 5	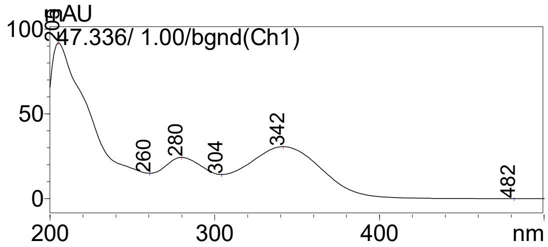

<table>
<tr><td>Flavonoid 6</td><td>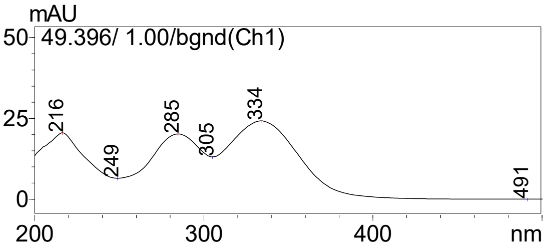
</td></tr>
<tr><td>Flavonoid 7</td><td>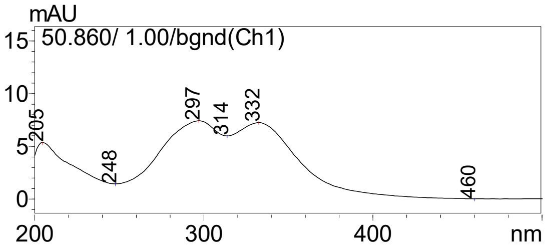
</td></tr>
<tr><td>Flavonoid 8</td><td>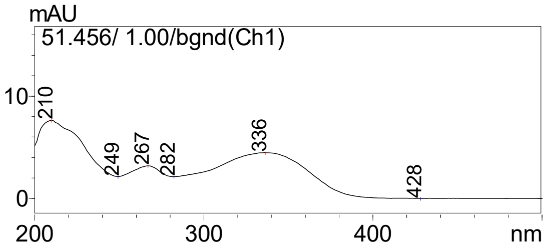
</td></tr>
<tr><td>Rosmarinic acid</td><td>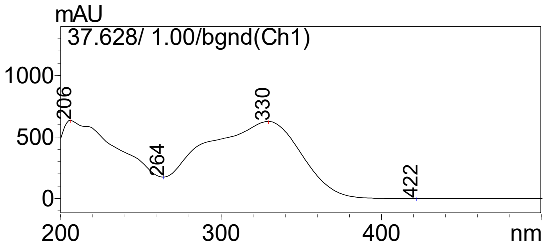
</td></tr>
</table>

Table S2 UV Spectra of compounds identified with HPLC analysis of rosemary extract

Carnosic acid	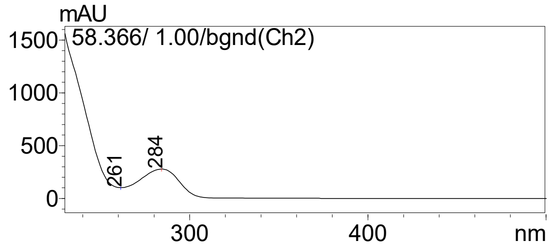
Carnosol	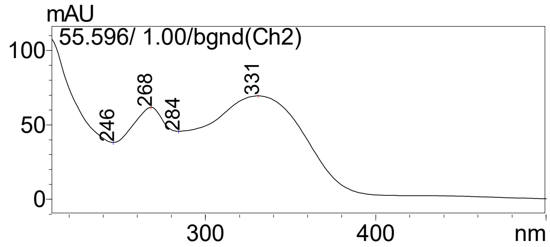
Flavonoid 1	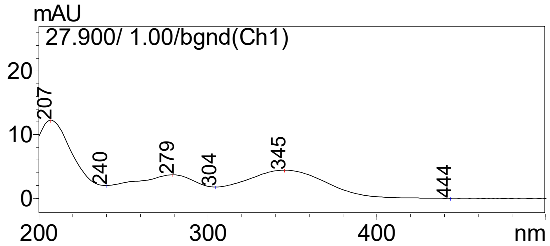
Flavonoid 2	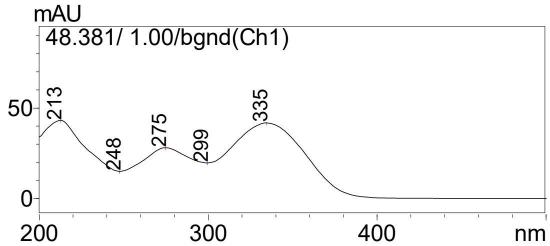
Flavonoid 3	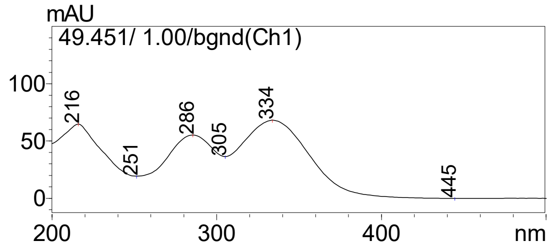
Flavonoid 4	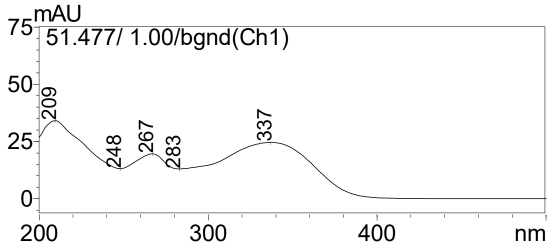
Flavonoid 5	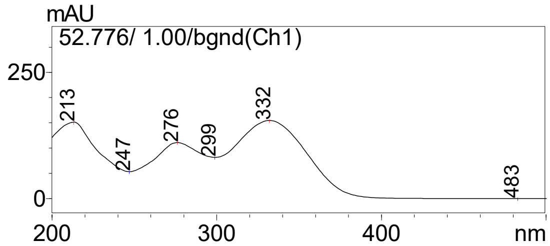

Flavonoid 6	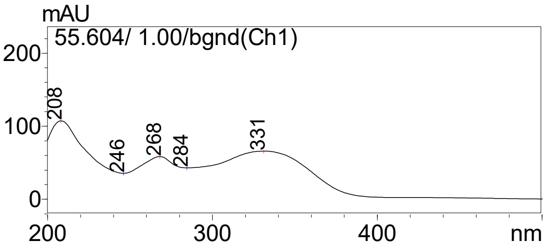
Rosmarinic acid	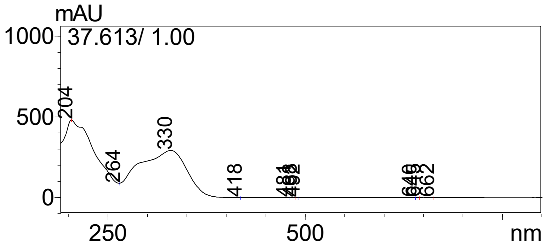
Rutin	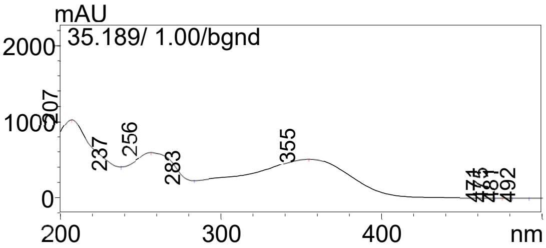

Table S3 UV Spectra of compounds identified with HPLC analysis of hypericum extract

Compound	UV Spectrum
Flavonoid 1	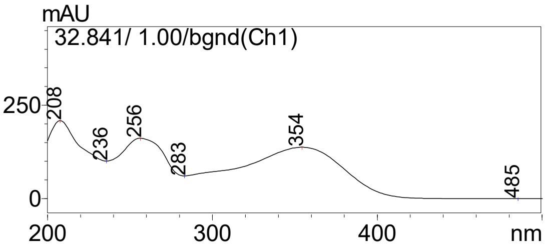
Flavonoid 2	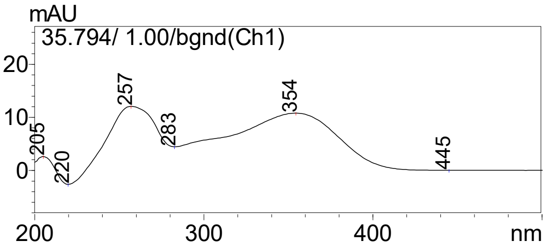
Flavonoid 3	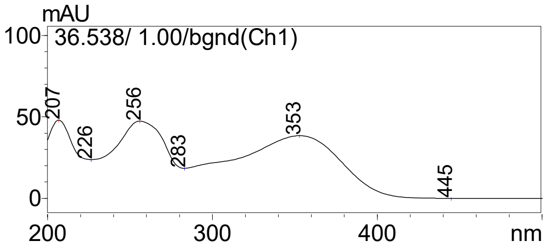
Flavonoid 4	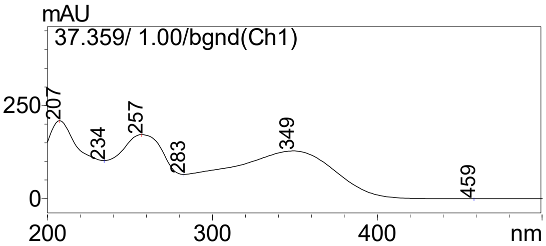
Flavonoid 5	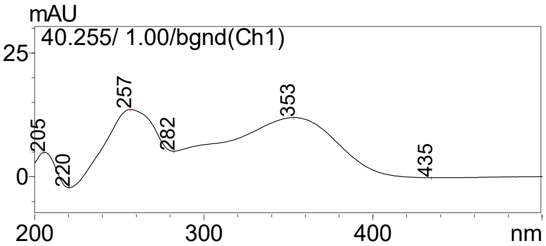
Flavonoid 6	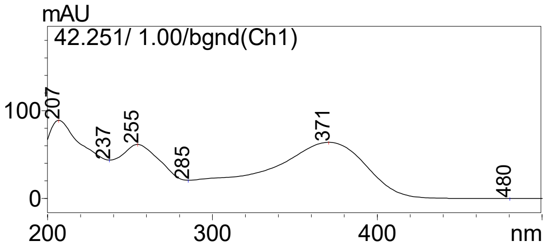

Flavonoid 7	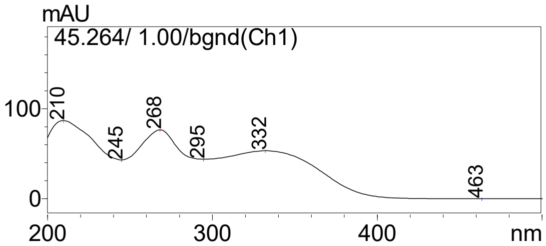
Hyperforin	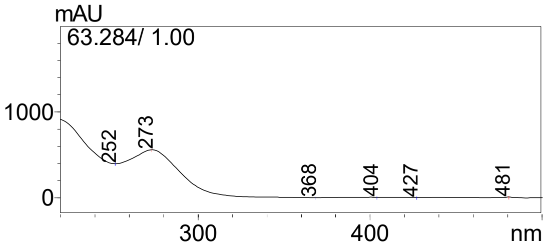
Hypericin	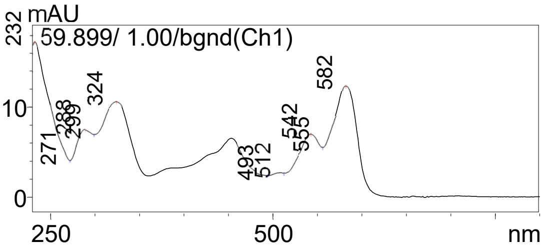

Table S4 UV Spectra of compounds identified with HPLC analysis of chamomile extract

Compound	UV Spectrum
Chamazulene	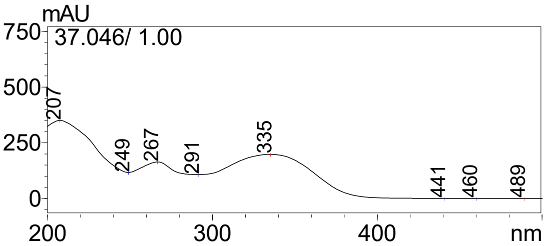
Flavonoid 1	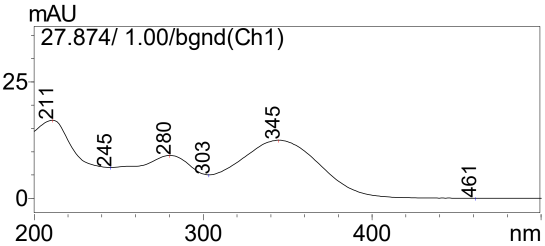
Flavonoid 2	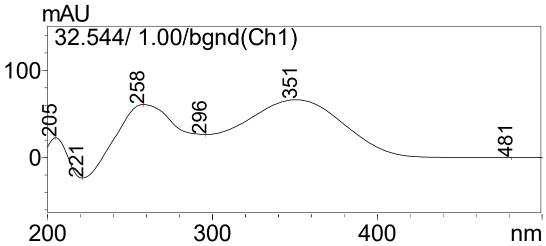
Flavonoid 3	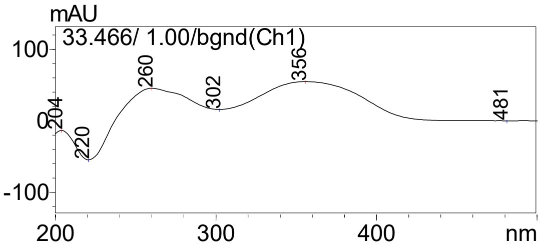
Flavonoid 4	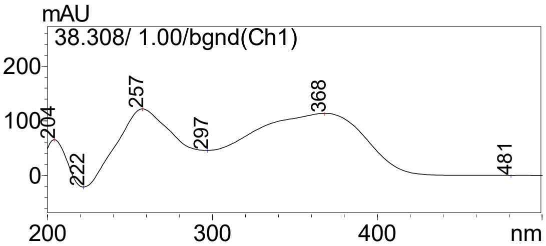
Flavonoid 5	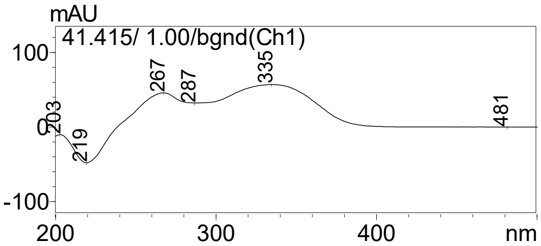

Flavonoid 6	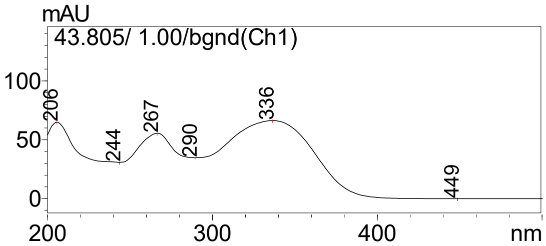
Flavonoid 7	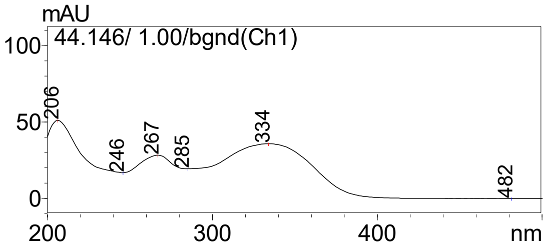
Flavonoid 8	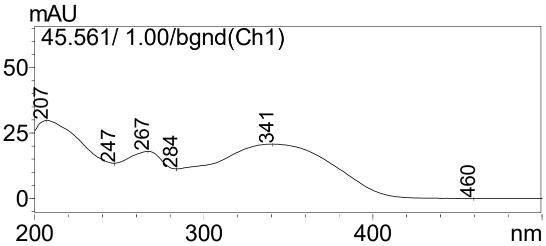
Phenolic acid 1	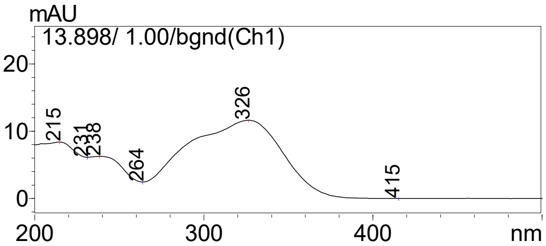
Phenolic acid 2	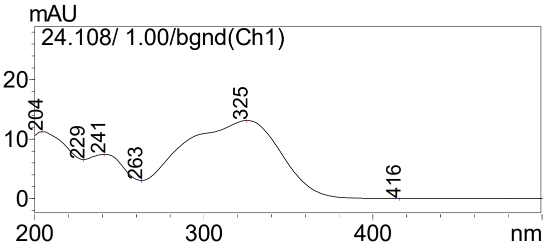
Phenolic acid 3	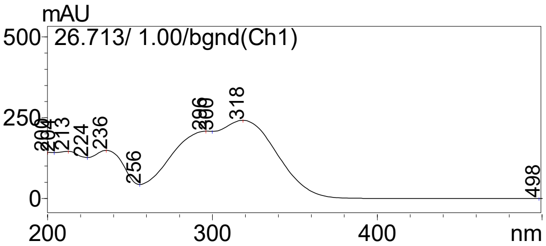
